# Bromocriptine improves glucose tolerance in obese mice via central dopamine D2 receptor-independent mechanism

**DOI:** 10.1371/journal.pone.0320157

**Published:** 2025-03-26

**Authors:** Hiroshi Tsuneki, Takahiro Maeda, Mayumi Takatsuki, Takahiro Sekine, Satsuki Masui, Kengo Onishi, Ryuta Takeda, Masanori Sugiyama, Takeshi Sakurai, Masashi Yanagisawa, Tsutomu Wada, Toshiyasu Sasaoka

**Affiliations:** 1 Department of Clinical Pharmacology, University of Toyama, Toyama, Japan; 2 Department of Integrative Pharmacology, University of Toyama, Toyama, Japan; 3 Faculty of Medicine/WPI-IIIS, University of Tsukuba, Tsukuba, Japan; Kyoto University Graduate School of Medicine, Kyoto, JAPAN

## Abstract

Bromocriptine, generally regarded as a dopamine D2 receptor agonist, has been used to treat patients with type 2 diabetes in the USA; however, its mechanisms of action including the receptors that mediate its anti-diabetic effects remain unclear. Therefore, we herein conducted pharmacological and genetic knockout experiments to investigate how bromocriptine improves glucose metabolism under type 2 diabetic conditions. Bromocriptine transiently increased blood glucose levels in both wild-type and dopamine D2 receptor-deficient mice. This glucose-elevating effect was blocked by the α2-adrenergic receptor antagonist yohimbine. On the other hand, when bromocriptine was administered daily for two weeks, glucose tolerance improved in wild-type mice fed a high-fat diet. Similar anti-diabetic effects of bromocriptine were observed in dopamine D2 receptor-deficient, dopamine D1 receptor-deficient, and orexin-deficient mice under the diet-induced obese condition as well as in genetically obese *db/db* mice. Bromocriptine-induced improvements in glucose tolerance were not affected by a pretreatment with the autonomic ganglion blocker hexamethonium, which suggested the involvement of the peripheral effects of bromocriptine. Given the biphasic properties of bromocriptine, we examined the drug effect on hepatic endoplasmic reticulum (ER) stress that dually regulates glucose metabolism. In the livers of diet-induced obese mice, the levels of ER stress markers, including C/EBP homologous protein (CHOP), were reduced by the daily administration of bromocriptine. In human hepatoma HepG2 cells, increases in CHOP expression by thapsigargin, a potent inducer of ER stress, were prevented by a pretreatment with low concentrations of bromocriptine, whereas high concentrations induced CHOP expression. These results suggest that low concentrations of bromocriptine caused beneficial ER stress preconditioning, which protected against subsequent severe ER stress in the liver. Therefore, bromocriptine may prevent obesity-induced glucose intolerance via peripheral mechanisms including promotion of hepatic ER homeostasis, but not central dopamine D2 receptor-mediated mechanisms.

## Introduction

A quick release (QR) formulation of bromocriptine mesylate (bromocriptine-QR) has been used to treat type 2 diabetes in the USA. Evidence indicates that bromocriptine-QR therapy for type 2 diabetes improves hyperglycemia and dyslipidemia without changes in body weight and also reduces the risk of cardiovascular disease [1–4]. Bromocriptine-QR is administered daily within 2 h of waking as chronotherapy because the central effects of bromocriptine in the early morning are considered to improve glucose homeostasis by resetting the circadian rhythm [1]. Bromocriptine also exerts sympatholytic effects that promote reductions in postprandial glucose level by inhibiting hepatic glucose production without increasing insulin secretion [2,4]. Since bromocriptine is often regarded as a dopamine D2 receptor agonist, the participation of dopamine D2 receptors in the regulation of glucose homeostasis has been proposed [5]. However, many types of receptors, including α2-adrenergic receptors, are involved in the effects of bromocriptine [6,7]. Therefore, the receptors that mediate its anti-diabetic effects have yet to be identified.

The beneficial long-term effects of bromocriptine on glucose tolerance have been demonstrated in animal models of diabetes [8–10]. The underlying mechanisms are considered to be associated with daily changes in dopamine and serotonin metabolism in the suprachiasmatic nucleus of the hypothalamus, which modulate the central circadian clock rhythm [11]. Circadian-timed administration of bromocriptine at the peak of daily central dopaminergic activity improved metabolic syndrome and vascular pathology in spontaneously hypertensive rats fed high-fat diet [12]. However, a recent study reported that neither circadian disruption nor modified treatment schedules abolished bromocriptine-induced improvements in glucose tolerance in diet-induced obese mice [13]. Moreover, bromocriptine acutely impaired glucose tolerance by suppressing insulin secretion in mice via a pancreatic α2-adrenergic receptor-dependent mechanism [7]. Until now, no genetic knockout study has been conducted to clarify whether dopamine D2 receptor mediates the acute glucose-elevating and/or chronic glucose-lowering effects of bromocriptine. In addition, it remains unknown whether the biphasic properties of bromocriptine underlie the mechanism of its anti-diabetic effect.

Although the central actions of bromocriptine have been reported to improve obesity-related metabolic disorders in rodents [1,8], emerging evidence indicates that bromocriptine can directly modulate the regulation of glucose metabolism in peripheral tissues, including the liver, white adipose tissue, skeletal muscle, and pancreatic islet α and β cells [14,15]. Moreover, recent high-throughput screening studies identified bromocriptine as the most effective compound to prevent endoplasmic reticulum (ER) dysregulation and cell death triggered by the thapsigargin (a sarco/endoplasmic reticulum Ca^2+^-ATPase inhibitor)-induced depletion of ER calcium in various cell lines and primary skeletal muscle cells [10,16]. Chronic ER stress in the liver causes hyperglycemia and glucose intolerance in obese mice [17]. However, to date, no study has examined the influence of bromocriptine on hepatic ER stress, except a study showing that higher concentrations of bromocriptine caused apoptosis in liver cancer cells [18]. It is well known that ER stress responses exhibited duality depending on the degree of input signal intensity and duration. Mild and severe ER stresses promote cell survival and death, respectively, and the former induces hormesis-like preconditioning to prevent the latter detrimental events [19,20]. Given the biphasic pharmacological properties of bromocriptine [7,13,21], we hypothesized that low concentrations of bromocriptine may promote preconditioning to prevent severe hepatic ER stress induced by nutrient overload under obese type 2 diabetic conditions.

In the present study, we conducted pharmacological and genetic knockout experiments to investigate whether the anti-diabetic effects of bromocriptine are mediated by dopamine D2 receptor or some other molecules/pathways relevant to the central regulation of glucose metabolism, such as, orexin, melatonin, leptin receptors, and the autonomic nervous system (ANS). Furthermore, we investigated whether and how bromocriptine prevents hepatic ER stress. The present study will provide insights into the mechanisms underlying the anti-diabetic effects of bromocriptine, which are complex and have yet to be clarified.

## Materials and methods

### Materials

Bromocriptine mesylate (026-18473, Fujifilm Wako, Osaka, Japan), atropine sulfate monohydrate (5908-99-6, Sigma-Aldrich, MO, USA), hexamethonium chloride dihydrate (081-04092, Wako Pure Chemical, Osaka, Japan), luzindole (15998, Cayman Chemical, Funakoshi Co., Tokyo, Japan), phenoxybenzamine hydrochloride (CA-305, Enzo Life Sciences, NY, USA), prazosin hydrochloride (15023, Cayman Chemical, Funakoshi Co.), propranolol hydrochloride (167-11593, Fujifilm Wako), yohimbine hydrochloride (259-00451, Fujifilm Wako), thapsigargin (209-17281, Fujifilm Wako), and cyclopiazonic acid (030-17171, Fujifilm Wako) were used.

### Animals

Male mice were examined in the present study. C57BL/6J mice were purchased from Japan SLC (Shizuoka, Japan). C57BLKS/J Iar-+Leprdb/+Leprdb (*db/db*) were purchased from the Institute for Animal Reproduction (Ibaraki, Japan). Dopamine D2 receptor knockout, dopamine D1 receptor knockout, and orexin knockout mice were prepared as previously described [22–24]. Mice were maintained at 20–26 °C under a standard 12-h light/dark cycle (i.e., light phase: zeitgeber time (ZT) 0–12, dark phase: ZT12-24) in the research animal facility of the University of Toyama. Mice were typically fed a normal chow diet (NCD, PicoLab Rodent Diet 20, PMI Nutrition International, USA). Diet-induced obesity was caused by feeding a 60 kcal% high-fat diet (HFD, D12492, Research Diets, New Brunswick, NJ) for 8 weeks. Mice were sacrificed by cervical dislocation for tissue isolation. Tail snip was conducted under halothane anesthesia to obtain blood samples used for the measurement of insulin levels. Animal study protocols were approved by the Committee of Animal Experiments at the University of Toyama (No. A2012PHA-6, A2015PHA-8, A2015PHA-19, A2018PHA-19, A2018PHA-21, A2021PHA-16, and A2021PHA-13), and all efforts were made to minimize suffering in the animal experiments.

### Measurements of blood glucose and serum insulin levels

In glucose tolerance tests, mice fasted for 6–16 h were intraperitoneally (i.p.) injected with glucose (0.5 or 2 g/kg) or saline. In insulin tolerance tests, mice fasted for 2–6 h were injected with insulin (Humulin R, a recombinant human insulin provided by Eli Lilly Japan, Kobe, Japan; 1 or 4 unit/kg, i.p.) or saline. Blood samples were obtained from the tail vein and blood glucose levels were measured using an Accu-Chek Aviva Nano glucose meter plus Test Strip F (Roche, Basel, Switzerland). In the analysis of glucose-stimulated insulin secretion, mice fasted for 6–16 h were injected with glucose (0.5, 1, or 2 g/kg, i.p.). Blood samples were collected from the tail vein, kept at 4^o^C overnight, and centrifuged at 3,000 rpm at 4^o^C for 15 min to prepare serum samples. Serum levels of insulin were analyzed with mouse insulin ELISA kits (MS302, Morinaga Institute of Biological Science, Yokohama, Japan; 296-89801, Fujifilm Wako) and SpectraMax i3 multi-mode microplate reader (Molecular Devices Japan, Tokyo, Japan), according to the manufacturer’s instructions.

### Measurement of locomotor activity

Changes in spontaneous locomotor activity were analyzed using the SCANET system (MV-40, Melquest, Toyama, Japan) at 5-min intervals for 180 min after an i.p. injection of bromocriptine or vehicle into wild-type and dopamine D2 receptor knockout mice.

### Experimental design for *in vivo* analyses

In experiments to examine the acute effects of bromocriptine on locomotor activity, dopamine D2 receptor knockout mice and their wild-type counterparts (4–5 months old) were administered bromocriptine (10 mg/kg, i.p.) or vehicle (10% ethanol, i.p.) under random fed conditions. To examine the acute effects of bromocriptine on blood glucose levels, C57BL/6J mice (8–10 weeks old or 4 months old) and dopamine D2 receptor knockout mice and their wild-type mice (8–9 months old) were administered bromocriptine (10 mg/kg, i.p.) or vehicle (10% ethanol, i.p.) under 2-h fasting conditions. Liver tissues were isolated under random fed conditions 1 h after the injection of bromocriptine or vehicle (10% ethanol). To investigate the mechanisms underlying the pharmacological effects of bromocriptine, hexamethonium (30 mg/kg), prazosin (2 mg/kg), yohimbine (2 mg/kg), atropine (2 mg/kg), or propranolol (5 mg/kg) in combination with or without phenoxybenzamine (5 mg/kg) was i.p. injected 15 min before the administration of bromocriptine or vehicle. All drugs were administered at 0.1 mL/10 g body weight. The concentrations of the drugs used were selected according to previous studies [25–29].

The long-term effects of bromocriptine were examined, as follows: C57BL/6J (8 weeks old), dopamine D2 receptor knockout (10 weeks old), dopamine D1 receptor knockout (3 months old), and orexin knockout (10 weeks old) mice were fed HFD for 8 weeks, and *db/db* mice (4 months old) were fed NCD. Bromocriptine (10 mg/kg, i.p.) or vehicle (10% ethanol, i.p.) was then injected daily at ZT14 for more than 2 weeks. Glucose tolerance tests (2 g glucose/kg, i.p. under 6-h fasting conditions) and insulin tolerance tests (1 unit insulin/kg, i.p. under 2-h fasting conditions) were conducted at ZT8, except for *db/db* mice. In *db/db* mice, glucose tolerance tests (0.5 g glucose/kg, i.p. under 16-h fasting conditions) and insulin tolerance tests (4 units insulin/kg, i.p. under 6-h fasting conditions) were conducted at ZT6. In the analysis of glucose-stimulated insulin secretion, C57BL/6J mice fasted for 6 h were injected with glucose (2 g/kg, i.p.), orexin knockout mice fasted for 6 h were injected with glucose (1 g/kg, i.p.), and *db/db* mice fasted for 16 h were injected with glucose (0.5 g/kg, i.p.). The liver tissues of *db/db* mice were isolated under 16-h fasting conditions. To investigate the mechanisms responsible for the effects of bromocriptine, yohimbine (3 mg/kg, subcutaneous injection), luzindole (2 mg/kg, i.p.), or hexamethonium (30 mg/kg, i.p.) was injected 15 min before the daily administration of bromocriptine or vehicle. Drug concentrations were selected according to previous studies [30–33].

The effects of bromocriptine against hepatic ER stress were investigated according to previous studies [34,35], as follows: C57BL/6J mice (7–8 weeks old) were fed HFD or NCD for 8 weeks, and bromocriptine (10 mg/kg, i.p.) or vehicle (10% ethanol, i.p.) was then administered daily at ZT14 for 2 weeks under random fed conditions, except on the last day. On the last day, mice were fasted for 24 h, administered bromocriptine (10 mg/kg, i.p.) or vehicle (10% ethanol, i.p.) at ZT14, and then refed HFD for 2 h to induce potent ER stress in the liver. Liver tissues were isolated at ZT16 and subjected to Western blot analysis.

### Cell culture

Human hepatocellular carcinoma HepG2 cells (JCRB1054) were provided by the Japanese Collection of Research Bioresources Cell Bank (National Institutes of Biomedical Innovation, Health and Nutrition, Osaka, Japan). This cell line was selected because the pharmacological/toxicological profiles of bromocriptine and thapsigargin have already been reported [18,36]. HepG2 cells were cultured in Dulbecco’s Modified Eagle Medium with 3.7 g/L NaHCO_3_ (31600034, Thermo Fisher Scientific, MA, USA) containing 10% fetal bovine serum (173012, Nichirei Biosciences, Tokyo, Japan), 100 units/mL penicillin (Meiji Seika, Tokyo, Japan), and 100 μg/mL streptomycin (Meiji Seika) at 37^o^C in a humidified atmosphere of 5% CO_2_. In pharmacological assays, HepG2 cells passaged 13–33 times were seeded at a density of 5.0 ×  10^5^ cells/well in 6-well plates (130184, Thermo Fisher Scientific) and incubated for 24 h. Cells were exposed to a 28-h treatment with vehicle [0.05% dimethyl sulfoxide (DMSO)] or bromocriptine (5 μM) or a 24-h vehicle incubation plus a 4-h treatment with thapsigargin (100 nM). To investigate the interaction of these two drugs, HepG2 cells were pretreated with bromocriptine (1–10 μM) or vehicle (0.05% DMSO) for 24 h, and thapsigargin (100 nM, an ER stress inducer) was then added for 4 h. Similarly, the interaction of bromocriptine and another ER stress inducer, cyclopiazonic acid (CPA, 50 μM) [37], was investigated in the same manner as above, but replacing thapsigargin with CPA. The concentrations of these drugs were selected according to previous studies [10,18,19]. They were then subjected to a Western blot analysis as described below.

### Western blot analysis

Liver tissues dissected from mice and HepG2 cells were stored at −80 °C until used. Protein extraction, sodium dodecyl sulfate polyacrylamide gel electrophoresis, and Western blotting were performed as previously described [38]. Proteins transferred to Immobilon P polyvinylidene fluoride membranes (IPVH00010, Merck Millipore, MA, USA) were blotted with primary and secondary antibodies and detected using the Chemi-Lumi One L kit (07880-70, Nacalai, Kyoto, Japan) and ImageQuant LAS4000EPUVmini (GE Healthcare Japan, Tokyo, Japan). The antibodies used were as follows: mouse anti-β-actin (8H10D10) monoclonal antibody (#3700, RRID:AB_2242334, Cell Signaling Technology, MA, USA, 1:2000 dilution), mouse anti-C/EBP homologous protein (CHOP) (L63F7) monoclonal antibody (#2895, RRID:AB_2089254, Cell Signaling Technology, 1:1000 dilution), rabbit anti-α-tubulin (11H10) monoclonal antibody (#2125, RRID:AB_2619646, Cell Signaling Technology, 1:2000 dilution), rabbit anti-eukaryotic initiation factor-2α (eIF2α) polyclonal antibody (#9722, RRID:AB_2230924, Cell Signaling Technology, 1:1000 dilution), rabbit anti-phospho-eIF2α (Ser51) polyclonal antibody (#9721, RRID:AB_330951, Cell Signaling Technology, 1:200 dilution), rabbit anti-inositol-requiring enzyme-1α (IRE1α) monoclonal antibody (#3294, RRID:AB_823545, Cell Signaling Technology, 1:1000 dilution), rabbit anti-phospho-IRE1 (Ser724) polyclonal antibody (ab48187, RRID:AB_873899, Abcam, Cambridge, UK, 1:500 dilution), rabbit anti-c-Jun N-terminal kinase (JNK) polyclonal antibody (sc-571, RRID:AB_632385, Santa Cruz Biotechnology, TX, USA, 1:1000 dilution), rabbit anti-phospho-SAPK/JNK (Thr183/Tyr185) polyclonal antibody (#9251, RRID:AB_331659, Cell Signaling Technology, 1:1000 dilution), anti-phosphoenolpyruvate carboxykinase 1 (PEPCK1) antibody (#12940, D12F5, RRID:AB_2687968, Cell Signaling Technology, 1:1000 dilution), donkey anti-rabbit IgG polyclonal secondary antibody-horseradish peroxidase (HRP) (NA934, RRID:AB_772206, Cytiva, 1:2000 dilution), sheep anti-mouse IgG monoclonal secondary antibody-HRP (NA931, RRID:AB_772210, Cytiva, 1:2000 dilution), and goat anti-mouse IgG polyclonal secondary antibody-HRP (A16066, RRID:AB_2534739, Invitrogen, Thermo Fisher Scientific, 1:2000 dilution). Antibodies were diluted with Can Get Signal Solution 1 & 2 (NKB-101, Toyobo, Osaka, Japan). Chemiluminescence images were analyzed using ImageJ software (National Institutes of Health, USA). The target protein level was normalized to the total expression level of the indicated protein in each cell and then averaged.

### Reverse transcription (RT)-quantitative polymerase chain reaction (qPCR)

RT-qPCR was conducted as previously described [38]. In brief, liver tissues dissected from mice were frozen in liquid nitrogen and stored at −80^o^C until used. RNA was extracted using TRIsure (BIO-38032, Nippon Genetics, Tokyo, Japan) and subjected to RT using the PrimeScript RT reagent Kit (RR037A, Takara Bio, Shiga, Japan). A qPCR analysis was then performed using TB Green Premix Ex Taq II (RR820L, Takara Bio) and the Mx3000P system (Stratagene, Agilent Technologies, CA, USA) with 40 cycles at 95^o^C for 10 s, 62^o^C for 20 s, and 72^o^C for 15 s. Mouse PCR primer pairs used in the present study were as follows: phosphoenolpyruvate carboxykinase (*Pepck*)-forward: 5’-ACTGACCCGGAACTTGGACA-3’, *Pepck*-reverse: CTGGTAGGTGGGCATGATTTG, glucose-6-phosphatase (*G6Pase*)-forward: GAAAAAGCCAACGTATGGATTCC, *G6Pase*-reverse: CAGCAAGGTAGATCCGGGA, *Chop*-forward: CCACCACACCTGAAAGCAGAA, *Chop*-reverse: AGGTGAAAGGCAGGGACTCA, *18S*-forward: GTAACCCGTTGAACCCCATT, *18S*-reverse: CCATCCAATCGGTAGTAGCG, S18-forward: AGTTCCAGCACATTTTGCGAG, and S18-reverse: TCATCCTCCGTGAGTTCTCCA. The expression level of the target PCR product was normalized to that of the housekeeping gene (18S rRNA or the *S18* ribosomal protein gene) in each cell and then averaged.

### Statistical analysis

Data are expressed as the means ±  standard deviation (S.D.). The two-tailed unpaired Student’s *t*-test was used to evaluate the significance of differences between two groups. Differences between three groups and those among more than four groups were evaluated by a one-way analysis of variance (ANOVA) with Dunnett’s multiple comparison test. Differences among four groups were evaluated by a two-way ANOVA with Tukey’s multiple comparison test, unless otherwise indicated. GraphPad Prism 9 software (GraphPad Software Inc., CA, USA) was used for statistical analyses. *P* values less than 0.05 were considered to be significant.

## Results

### Bromocriptine bidirectionally regulates glucose metabolism independently of dopamine D2 receptors

**Fig 1:**
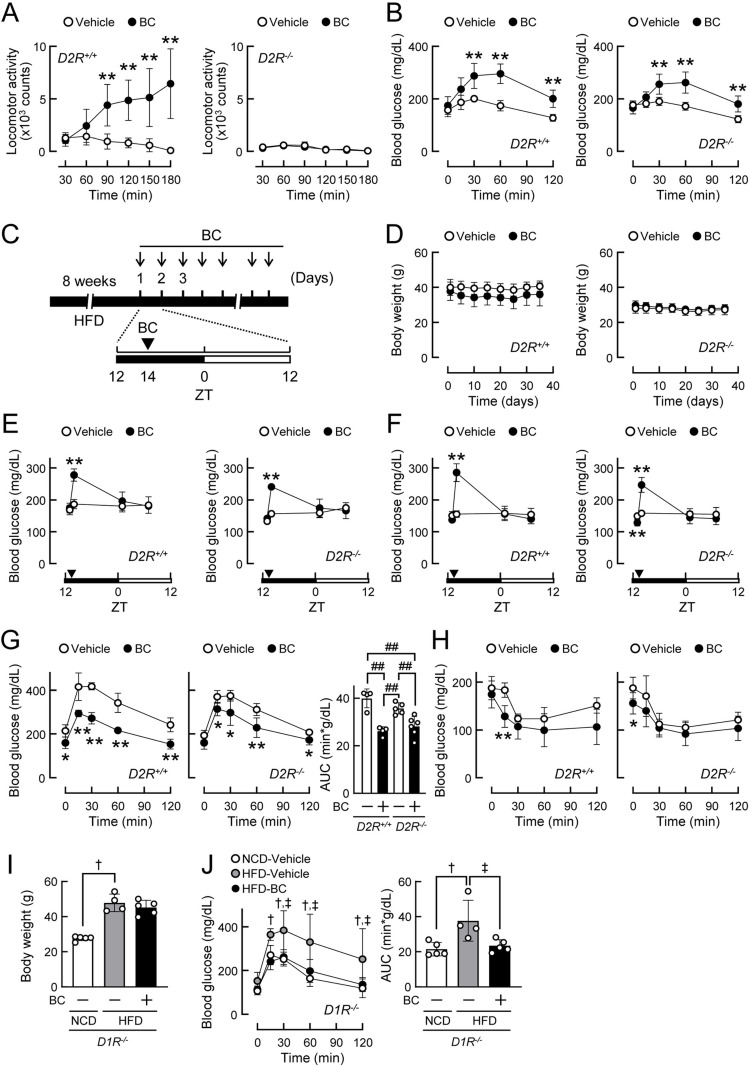
Biphasic effects of bromocriptine on blood glucose levels via the dopamine D2 receptor-independent mechanism. (A-B) Acute effects of bromocriptine (BC) in dopamine D2 receptor knockout (*D2R*^*-/-*^) and wild-type (*D2R*^*+/+*^) mice. Mice were injected with BC (10 mg/kg, i.p.) or vehicle (10% ethanol). (A) Effects of BC on locomotor activity in *D2R*^*+/+*^ (left) and *D2R*^*-/-*^ mice (right) at 4–5 months of age. n = 5 per group. (B) Effects of BC on blood glucose levels under 2-h fasting conditions in *D2R*^*+/+*^ (left) and *D2R*^*-/-*^ mice (right) at 8–9 months of age. n = 5 per group. (C-H) Long-term effects of BC on glucose metabolism in *D2R*^*+/+*^ and *D2R*^*-/-*^ mice fed a high-fat diet (HFD) for 8 weeks (from 10 weeks of age). HFD-fed *D2R*^*+/+*^ and *D2R*^*-/-*^ mice were administered BC (10 mg/kg, i.p.) or vehicle daily at ZT14 for 2 weeks. n = 4–6 per group. (C) Timeline of the experimental procedure. (D) Body weights in *D2R*^*+/+*^ (left) and *D2R*^*-/-*^ mice (right). (E-F) Random fed blood glucose levels just after the injection of BC at ZT14 on day 1 (E) and day 10 (F). (G) Glucose tolerance test in *D2R*^*+/+*^ (left) and *D2R*^*-/-*^ mice (middle) treated daily with BC or vehicle for 2 weeks. The right panel shows the glucose area under the curves (AUC) calculated on the left and middle panels. (H) Insulin tolerance test in *D2R*^*+/+*^ (left) and *D2R*^*-/-*^ mice (right) treated daily with BC or vehicle for 2 weeks. (I-J) Long-term effects of BC on glucose metabolism in dopamine D1 receptor knockout (*D1R*^*-/-*^) mice fed HFD or a normal chow diet (NCD) for 8 weeks (from 3 months of age). Mice were administered BC (10 mg/kg, i.p.) or vehicle daily at ZT14 for 2 weeks. n = 4–5 per group. (I) Body weights after 2 weeks of drug administration. (J) The glucose tolerance test conducted after 2 weeks of drug administration. Values are expressed as the means ±  S.D. * *p* < 0.05 and **p < 0.01 by the Student’s *t*-tes*t*. ^##^*p* < 0.01 by a two-way ANOVA with Tukey’s test. ^†^*p* < 0.05 (vehicle-treated mice fed NCD vs. vehicle-treated mice fed HFD) and ^‡^*p* < 0.05 (vehicle-treated mice fed HFD vs. BC-treated mice fed HFD) by a one-way ANOVA with Dunnett’s test.

Locomotor activity was increased by the administration of bromocriptine (10 mg/kg) in wild-type, but not dopamine D2 receptor knockout mice (Fig 1A). In contrast, when wild-type and dopamine D2 receptor knockout mice maintained on NCD were treated with bromocriptine (10 mg/kg), acute and transient increases in blood glucose levels were observed in both genotypes (Fig 1B). These results indicate that bromocriptine increased physical activity in a dopamine D2 receptor-dependent manner, whereas it elevated blood glucose levels in a dopamine D2 receptor-independent manner.

To examine the effects of bromocriptine under type 2 diabetic conditions, wild-type and dopamine D2 receptor-deficient mice were fed HFD for 8 weeks, and bromocriptine (10 mg/kg) was then administered daily at the beginning of the awake phase (ZT14) for 2 weeks (Fig 1C). Body weights were not affected by the daily administration of bromocriptine (Fig 1D). On days 1 and 10, bromocriptine acutely increased blood glucose levels in both wild-type and dopamine D2 receptor knockout mice (Fig 1E and 1F). After a two-week treatment with bromocriptine, glucose tolerance improved in both wild-type and dopamine D2 receptor knockout mice (Fig 1G), whereas insulin tolerance remained unchanged (Fig 1H). These results indicate that bromocriptine exerted bidirectional effects (i.e., early-onset glucose-elevating and late-onset glucose-lowering effects) in a dopamine D2 receptor-independent manner.

In dopamine D1 receptor knockout mice fed HFD, the daily administration of bromocriptine did not affect body weight (Fig 1I), but improved glucose tolerance, resulting in a decrease in blood glucose to normal levels during the glucose tolerance test (Fig 1J). Therefore, dopamine D1 receptors also did not mediate the improvements achieved in glucose tolerance with the daily administration of bromocriptine under diet-induced obese conditions.

### Bromocriptine acutely increases blood glucose levels via α2-adrenergic receptors

**Fig 2:**
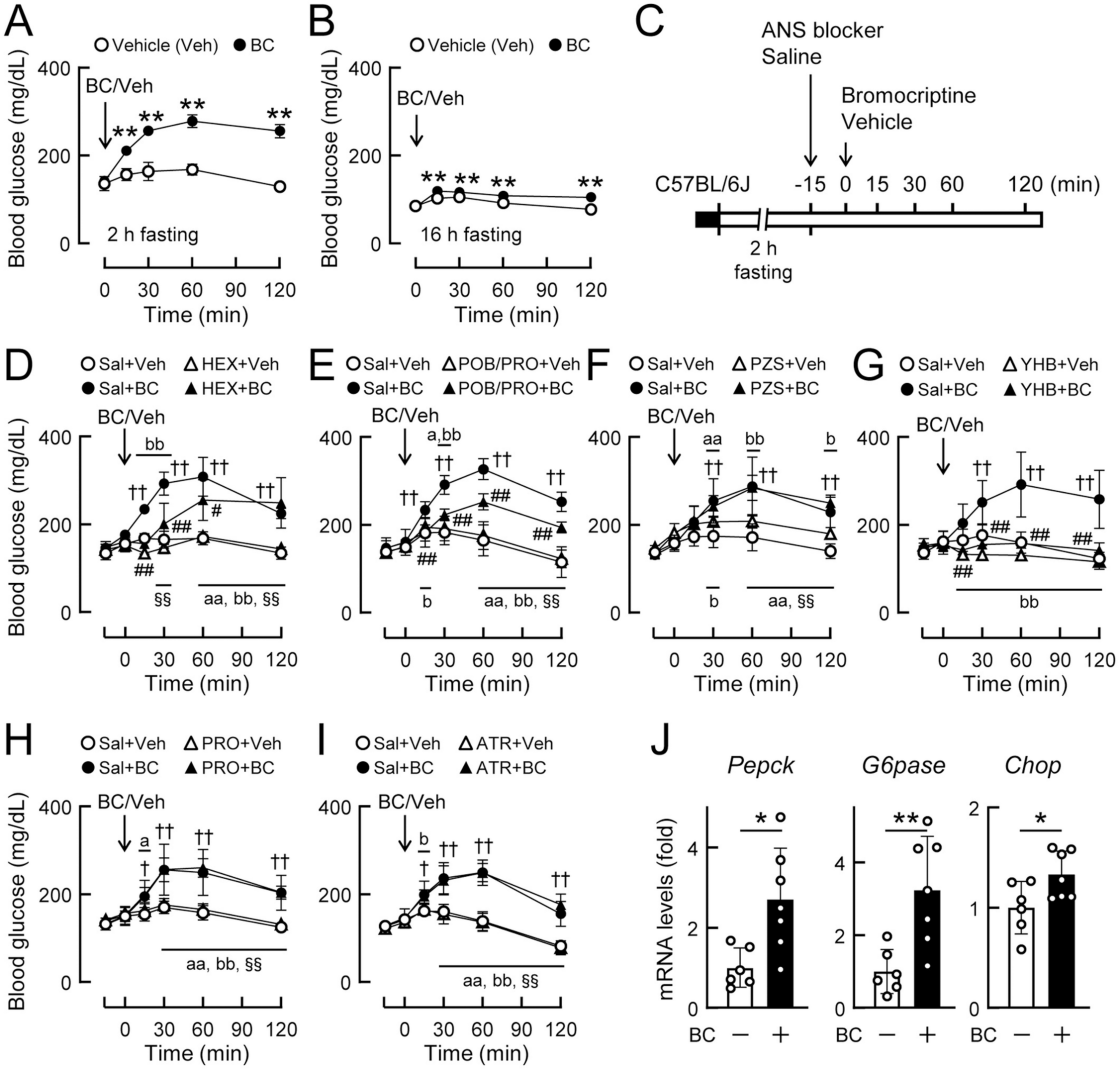
Acute effects of bromocriptine on blood glucose levels via an **α2-adrenergic receptor-dependent mechanism.** (A-B) Blood glucose levels just after the injection of bromocriptine (BC, 10 mg/kg, i.p.) or vehicle (Veh, 10% ethanol) in 4-month-old C57BL/6J mice under 2-h (A) and 16-h fasting conditions (B). n = 5 per group. (C-I) Effects of a pretreatment with autonomic nervous system (ANS)-blocking agents on the acute glucose-elevating effects of BC in C57BL/6J mice (2–3 months old). (C) Experimental procedures. (D-I) Effects of a pretreatment with hexamethonium (D, HEX), a cocktail of phenoxybenzamine and propranolol (E, POB+PRO), prazosin (F, PZS), yohimbine (G, YHB), propranolol alone (H), atropine (I, ATR), or saline (Sal) on blood glucose levels in mice treated with BC or vehicle under 2-h fasting conditions. n = 4–5 per group. (J) Effects of BC on the expression levels of gluconeogenesis markers (*Pepck* and *G6pase* mRNAs) and ER stress marker (*Chop* mRNA) in the livers of C57BL/6J mice (8 weeks old) fed *ad libitum*. Liver tissues were isolated 1 h after the injection of BC (10 mg/kg, i.p.) or vehicle at ZT14. n = 6–7 per group. Values are expressed as the means ±  S.D. Significant differences in panels A, B, and J were examined by the Student’s *t*-test: * *p* < 0.05 and **p < 0.01. Significant differences in panels D-I were assessed by a two-way ANOVA with Tukey’s test: ^†^*p* < 0.05 and ^††^*p* < 0.01 (saline +  vehicle treatment vs. saline +  BC treatment), ^#^*p* < 0.05 and ^##^*p* < 0.01 (saline +  BC treatment vs. blocker +  BC treatment), ^§§^*p* < 0.01 (blocker +  vehicle treatment vs. blocker +  BC treatment), ^a^*p* < 0.05 and ^aa^*p* < 0.01 (saline +  vehicle treatment vs. blocker +  BC treatment), and ^b^*p* < 0.05 and ^bb^*p* < 0.01 (saline +  BC treatment vs. blocker +  vehicle treatment).

To clarify the mechanisms responsible for the bidirectional effects of bromocriptine on glucose metabolism, we investigated the pharmacological profiles of acutely administered bromocriptine using C57BL/6J mice maintained on NCD. Bromocriptine (10 mg/kg, i.p.) did not affect the total 24-h food intake in mice that were fasted for 24 h (S1 Fig). Bromocriptine (10 mg/kg, i.p.) markedly increased blood glucose levels under 2-h fasting conditions (Fig 2A); however, its efficacy was diminished under 16-h fasting conditions (Fig 2B). Therefore, glucose/insulin deprivation appeared to disturb the glucose-elevating effects of bromocriptine. Serum insulin levels showed a decrease trend in bromocriptine-treated mice, compared to vehicle controls (S2 Fig). We also examined the involvement of the ANS in the acute effects of bromocriptine by using autonomic blocking agents (Fig 2C). When mice were pretreated with hexamethonium, an autonomic ganglionic blocker, the glucose-elevating effects of bromocriptine were partly reduced (Fig 2D). The cocktail of phenoxybenzamine (a non-selective antagonist of α-adrenergic receptors) and propranolol (a β-adrenergic receptor blocker) also partly inhibited the effects of bromocriptine (Fig 2E). Although prazosin, a selective α1-adrenergic receptor blocker, did not exert this effect (Fig 2F), yohimbine, a selective α2-adrenergic receptor blocker, completely inhibited the glucose-elevating effects of bromocriptine (Fig 2G). Neither propranolol alone nor atropine (a muscarinic acetylcholine receptor blocker) had an impact on the effects of bromocriptine on blood glucose levels (Fig 2H and 2I). In the liver, the expression levels of gluconeogenesis (*Pepck* and *G6pase* mRNA) and ER stress (*Chop* mRNA) markers were acutely elevated by bromocriptine (Fig 2J). These results indicate that bromocriptine acutely increased blood glucose levels in an α2-adrenergic receptor-dependent manner.

### Daily administration of bromocriptine improves glucose tolerance under type 2 diabetic conditions independently of the central nervous system (CNS) and ANS

**Fig 3:**
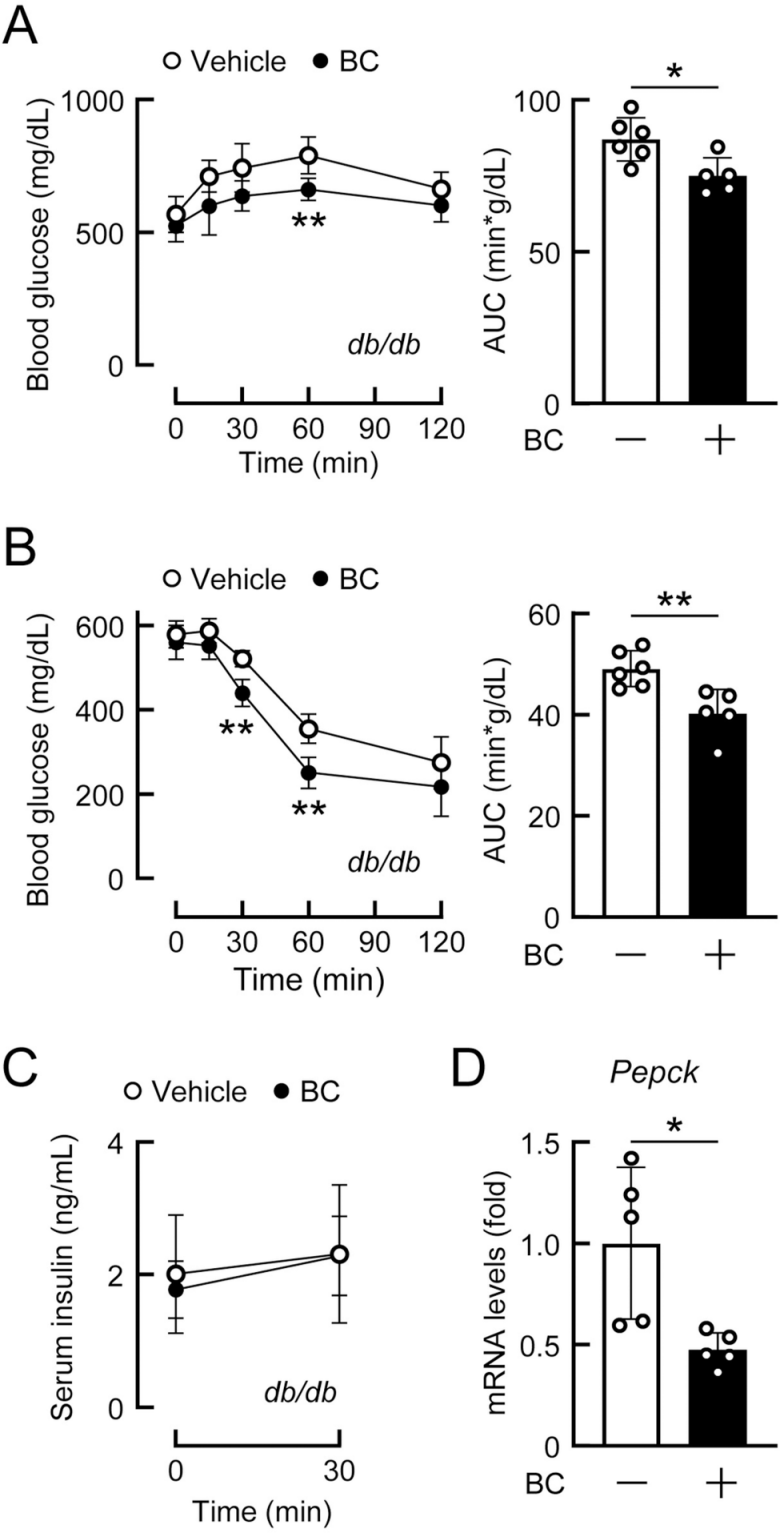
Daily administration of bromocriptine improved glucose metabolism in genetically-obese *db/db* mice. (A-D) Diabetic *db/db* mice (4 months old) fed a normal chow diet were administered bromocriptine (BC, 10 mg/kg, i.p.) or vehicle (10% ethanol) daily at ZT14 for 2 weeks. n = 5–6 per group. (A) The glucose tolerance test was conducted 2 weeks after the daily administration of BC. (B) The insulin tolerance test was conducted 2 weeks after the daily administration of BC. (C) Serum insulin levels after glucose loading. Glucose-stimulated insulin secretion was measured 2 weeks after the daily administration of BC. (D) The levels of *Pepck* mRNA, a gluconeogenesis marker, in the livers of mice treated daily with BC or vehicle for 4 weeks. Liver tissues were isolated under 16-h fasting conditions. Values are expressed as the means ±  S.D. * *p* < 0.05 and **p < 0.01 by the Student’s *t*-tes*t*.

We investigated the mechanisms underlying the long-term effects of bromocriptine on glucose metabolism under obese type 2 diabetic conditions, particularly those involving the CNS and ANS. In leptin receptor-deficient *db/db* mice fed NCD, the daily administration of bromocriptine improved glucose tolerance (Fig 3A) and increased insulin sensitivity (Fig 3B) without affecting glucose-induced insulin secretion (Fig 3C). Body weights were not affected by the administration of bromocriptine (vehicle-treated group: 33.9 ±  1.7 g, n = 6; bromocriptine-treated group: 34.6 ±  1.5 g, n = 5; *p* = 0.767). *Pepck* mRNA levels in the liver of *db/db* mice were reduced by the daily administration of bromocriptine (Fig 3D). Therefore, the long-term treatment with bromocriptine suppressed hepatic glucose production and improved glucose metabolism in a leptin receptor-independent manner.

**Fig 4:**
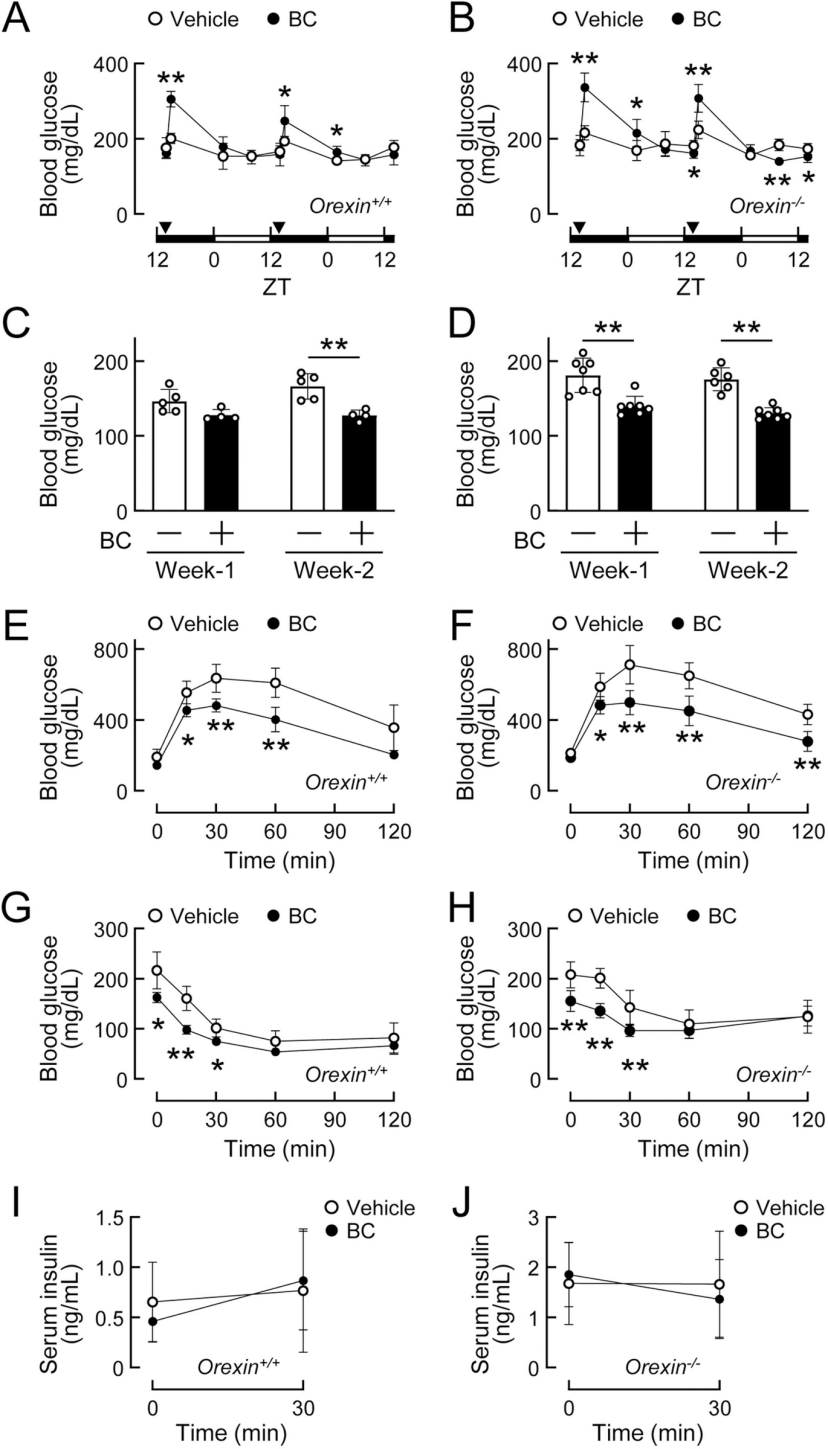
Daily administration of bromocriptine improved glucose metabolism in orexin knockout mice under diet-induced obese conditions. (A-J) Wild-type (*Orexin*^*+/+*^) and orexin knockout (*Orexin*^*-/-*^) mice at 10 weeks of age were fed a high-fat diet for 8 weeks, and bromocriptine (BC, 10 mg/kg, i.p.) or vehicle (10% ethanol) was then administered daily at ZT14 for 2 weeks. n = 4–7 per group. (A-B) Transient increases in blood glucose levels after the BC injection at ZT14 on day 1 and day 2 in *Orexin*^*+/+*^ (A) and *Orexin*^*-/-*^ mice (B). (C-D) Random fed blood glucose levels measured at ZT8 after 1 and 2 weeks of the BC treatment in *Orexin*^*+/+*^ (C) and *Orexin*^*-/-*^ mice (D). (E-F) The glucose tolerance test conducted 2 weeks after the daily administration of BC in *Orexin*^*+/+*^ (E) and *Orexin*^*-/-*^ mice (F). (G-H) The insulin tolerance test conducted 2 weeks after the daily administration of BC in *Orexin*^*+/+*^ (G) and *Orexin*^*-/-*^ mice (H). (I-J) Serum insulin levels during the glucose-stimulated insulin secretion test conducted 2 weeks after the daily administration of BC in *Orexin*^*+/+*^ (I) and *Orexin*^*-/-*^ mice (J). Values are expressed as the means ±  S.D. * *p* < 0.05 and **p < 0.01 by the Student’s *t*-tes*t*.

A hypothalamic neuropeptide orexin has been reported to exert biphasic changes in blood glucose levels, similar to bromocriptine [39]. To examine the involvement of the central orexin system in the effects of bromocriptine, wild-type (*Orexin*^*+/+*^) and orexin knockout (*Orexin*^*-/-*^) mice fed HFD for 8 weeks were treated daily with bromocriptine. Body weights were not affected by the drug treatment in *Orexin*^*+/+*^ (vehicle-treated group: 38.3 ±  2.4 g, n = 5; bromocriptine-treated group: 37.4 ±  3.7 g, n = 4; *p* = 0.840) or *Orexin*^*-/-*^ mice (vehicle-treated group: 46.5 ±  1.9 g, n = 7; bromocriptine-treated group: 47.0 ±  1.3 g, n = 7; *p* = 0.845). Blood glucose levels transiently increased after each injection of bromocriptine in *Orexin*^*+/+*^ and *Orexin*^*-/-*^ mice (Fig 4A and 4B). Fed blood glucose levels were reduced 2 weeks after the daily administration of bromocriptine in *Orexin*^*+/+*^ mice (Fig 4C), and 1 and 2 weeks after the daily administration of bromocriptine in *Orexin*^*-/-*^ mice (Fig 4D). The daily administration of bromocriptine improved glucose tolerance in both *Orexin*^*+/+*^ and *Orexin*^*-/-*^ mice (Fig 4E and 4F). Blood glucose levels during the insulin tolerance test were lowered by the bromocriptine treatment in both *Orexin*^*+/+*^ and *Orexin*^*-/-*^ mice (Fig 4G and 4H). Glucose-stimulated insulin secretion was not affected by the daily administration of bromocriptine in either mouse (Fig 4I and 4J). These results demonstrate that the central orexin system did not mediate bromocriptine-induced improvements in glucose metabolism under obese conditions.

**Fig 5:**
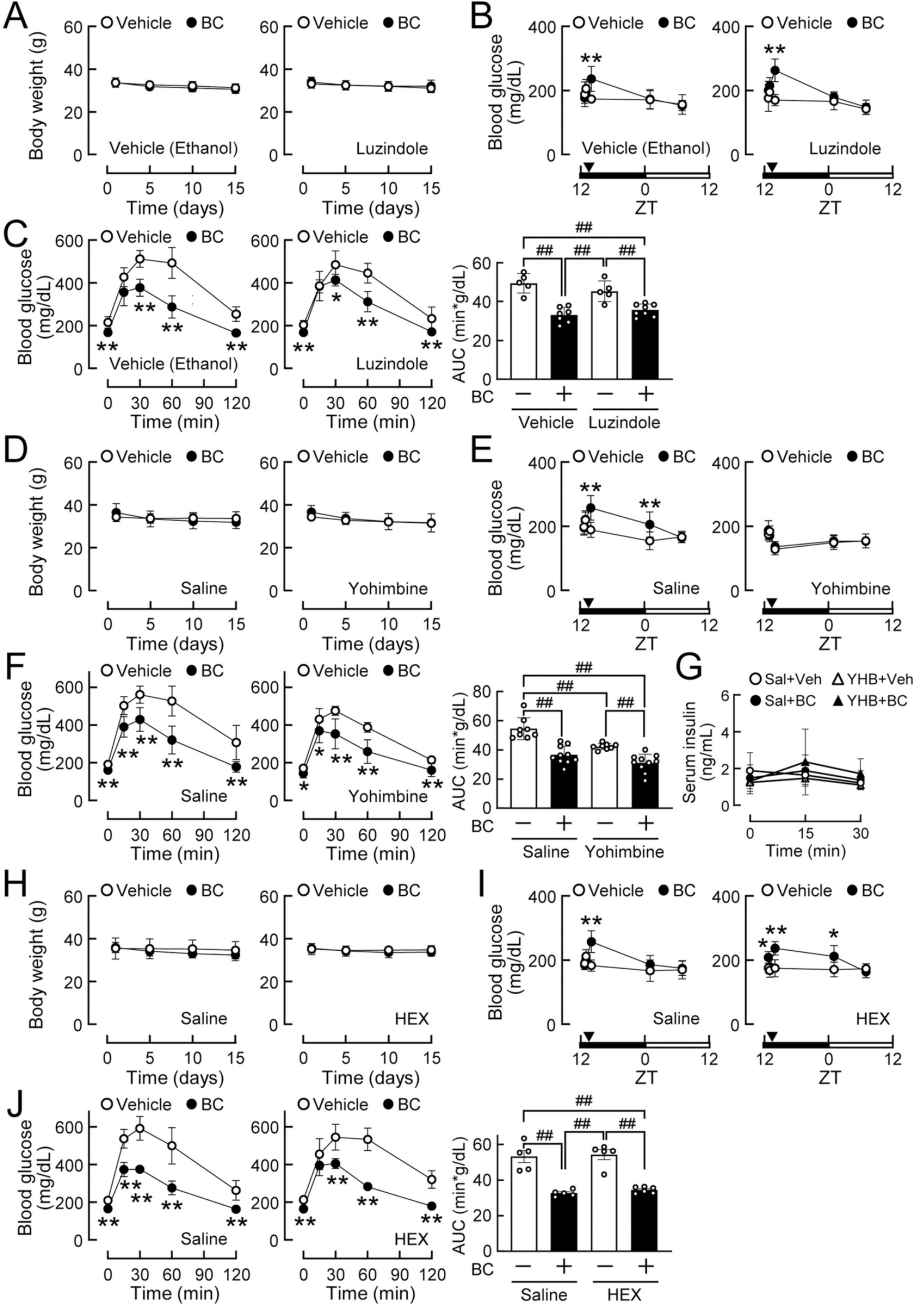
Daily administration of bromocriptine improved glucose tolerance in diet-induced obese mice independently of melatonin and the autonomic nervous system. C57BL/6J mice (8 weeks old) were fed HFD for 8 weeks. (A-C) Impact of a pretreatment with luzindole on the effects of bromocriptine (BC). Diet-induced obese mice were treated with luzindole (2 mg/kg, i.p.) or vehicle (1% ethanol, i.p.) and, 15 min later, BC (10 mg/kg, i.p.) or vehicle (10% ethanol, i.p.) was administered at ZT14. These drug treatments were repeated for 2 weeks. n = 5–8 per group. (A) Effects of BC or vehicle (10% ethanol) on body weights in mice pretreated daily with vehicle (left, 1% ethanol) or luzindole (right). (B) Transient increases in blood glucose levels by the BC injection in mice pretreated with vehicle (left) or luzindole (right) on day 1. (C) The glucose tolerance test conducted 2 weeks after the administration of BC to mice pretreated with vehicle (left) or luzindole (middle). The right panel shows the glucose area under the curve (AUC) calculated on the left and middle panels. (D-G) Impact of the pretreatment with yohimbine on the effects of BC. Diet-induced obese mice were treated with yohimbine (YHB, 3 mg/kg, s.c.) or vehicle (saline, Sal, s.c.) and, 15 min later, BC (10 mg/kg, i.p.) or vehicle (10% ethanol, i.p.) was administered at ZT14. These drug treatments were repeated for 2 weeks. (D) Effects of BC or vehicle on body weights in mice pretreated daily with saline (left) or yohimbine (right). n = 5–7 per group. (E) Transient increases in blood glucose levels by the BC injection in mice pretreated with saline (left) or yohimbine (right) on day 1. n = 8–10 per group. (F) The glucose tolerance test conducted 3 weeks after the administration of BC to mice pretreated with saline (left) or yohimbine (middle). The right panel shows glucose AUC based on the left and middle panels. n = 8–10 per group. (G) Serum insulin levels during the glucose-stimulated insulin secretion test conducted 4 weeks after the daily administration of BC and/or yohimbine. n = 5–7 per group. (H-J) Impact of the pretreatment with hexamethonium (HEX) on the effects of BC. Diet-induced obese mice were treated with HEX (30 mg/kg, i.p.) or vehicle (saline, i.p.) and, 15 min later, BC (10 mg/kg, i.p.) or vehicle (10% ethanol, i.p.) was administered at ZT14. These drug treatments were repeated for 2 weeks. n = 5–6 per group. (H) Effects of BC or vehicle on body weights in mice pretreated daily with saline (left) or HEX (right). (I) Transient increases in blood glucose levels by the BC injection in mice pretreated with saline (left) or HEX (right) on day 1. (J) The glucose tolerance test conducted 2 weeks after the administration of BC to mice pretreated with saline (left) or HEX (middle). The right panel shows glucose AUC calculated on the left and middle panels. Values are expressed as the means ±  S.D. * *p* < 0.05 and **p < 0.01 by the Student’s *t*-tes*t*. ^##^*p* < 0.01 by a two-way ANOVA with Tukey’s test.

To examine the involvement of the melatonin system in the effects of bromocriptine, mice fed HFD for 8 weeks were treated daily with bromocriptine in combination with or without luzindole, a selective melatonin receptor antagonist. Body weights were not affected by these drugs (Fig 5A). The transient increase in blood glucose levels just after the injection of bromocriptine was observed in the absence and presence of luzindole (Fig 5B). The improvement in glucose tolerance after the daily administration of bromocriptine was also noted in the presence of luzindole (Fig 5C). Therefore, the bidirectional effects of bromocriptine on glucose metabolism were independent of the melatonin system.

When mice fed HFD for 8 weeks were treated daily with bromocriptine in combination with or without yohimbine, body weights were not affected by the drug treatment (Fig 5D). The blood glucose elevation just after the bromocriptine injection was inhibited by yohimbine (Fig 5E), consistent with its effects in mice fed NCD (Fig 2G). In contrast, the improvement in glucose tolerance after the daily administration of bromocriptine was not affected by yohimbine in diet-induced obese mice (Fig 5F). These results indicate that α2-adrenergic receptors mediated the acute, but not long-term effects of bromocriptine on glucose metabolism. Glucose-stimulated insulin secretion was not affected by the daily administration of bromocriptine and/or yohimbine (Fig 5G), indicating that daily-administered bromocriptine improved glucose tolerance independently of insulin secretion.

The daily administration of bromocriptine in combination with or without hexamethonium did not affect body weights in diet-induced mice (Fig 5H). Under autonomic blockade with the hexamethonium pretreatment, the acute glucose-elevating effects of bromocriptine (Fig 5I) and improvements in glucose tolerance after the long-term treatment with bromocriptine (Fig 5J) were both detected, and both were also found in the absence of hexamethonium. Therefore, the daily administration of bromocriptine appeared to improve obesity-associated glucose intolerance through a mechanism downstream of the CNS and ANS.

### Daily administration of bromocriptine improves hepatic ER stress in obese mice

**Fig 6:**
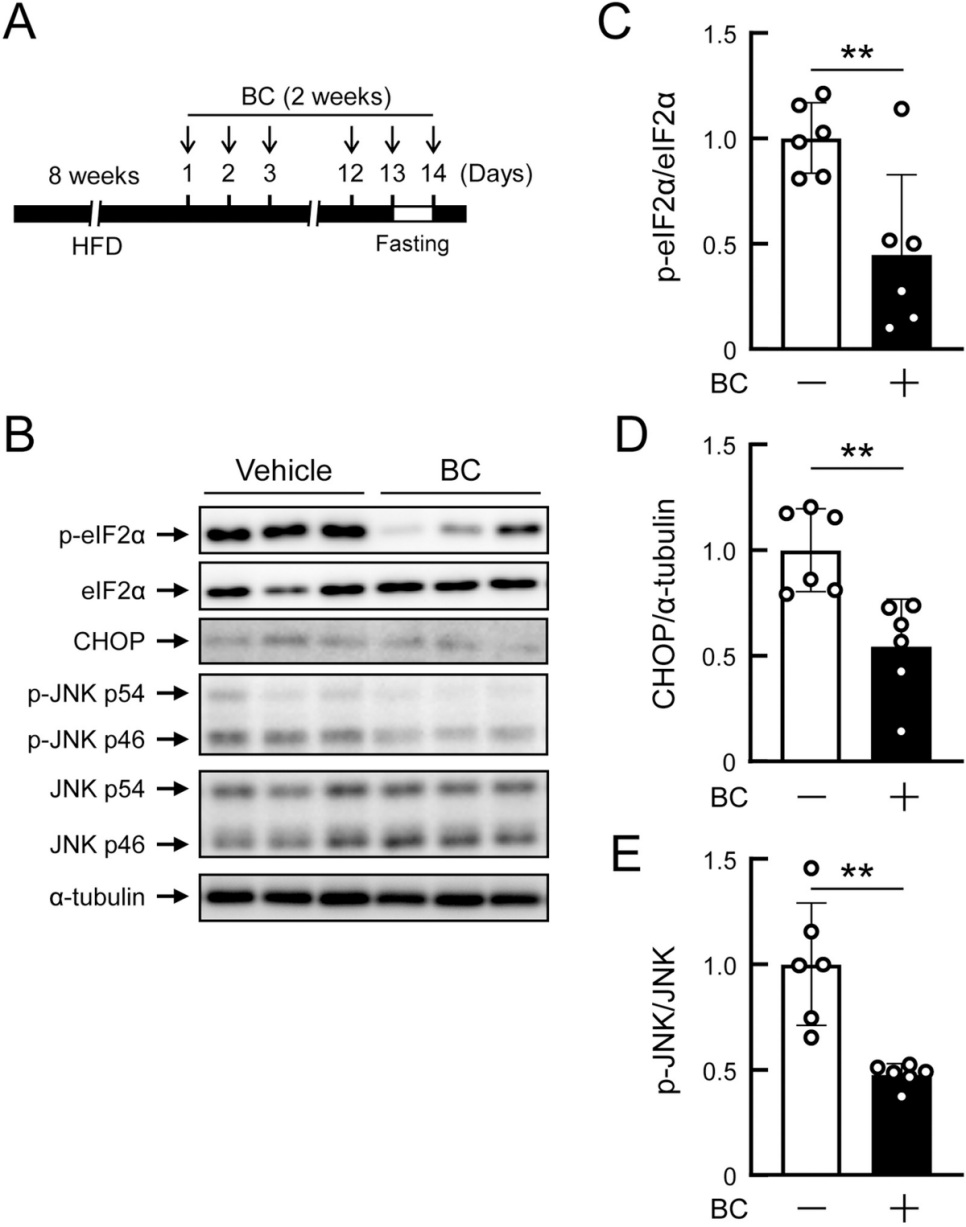
Daily administration of bromocriptine suppressed ER stress in livers of diet-induced obese mice. C57BL/6J mice (7–8 weeks old) were fed a high-fat diet for 8 weeks and bromocriptine (BC, 10 mg/kg, i.p.) or vehicle (10% ethanol) was then administered daily at ZT14 for 2 weeks. Liver tissues were isolated at ZT16 under pathophysiological ER stress condition (i.e., 2 h of refeeding after 24 h of fasting). n = 6 per group. (A) Experimental protocol. (B) Representative Western blot images. (C-E) Effects of BC on the levels of p-eIF2α/eIF2α, CHOP/α-tubulin, and p-JNK/JNK in the livers of mice treated with or without BC. Values are expressed as the means ±  S.D. **p < 0.01 by the Student’s *t*-*t*est.

As described above, some peripheral mechanism is predicted to be involved in bromocriptine-induced improvements in glucose metabolism. Reduction of hepatic glucose production has been regarded as the primary effect of the bromocriptine treatment [1]; however, the underlying mechanism remains unknown. Since hepatic ER stress is a main cause of hepatic glucose output [17] and because bromocriptine is a potent drug that modulates ER homeostasis [10], we examined the effects of bromocriptine on the expression levels of ER stress markers in the livers of mice fed HFD for 8 weeks. To evaluate the response to pathophysiological ER stress in the liver, mice were fasted for 24 h and then refed for 2 h (Fig 6A), according to a previously reported method [34, 35]. After the daily administration of bromocriptine for 2 weeks, the phosphorylation levels of eIF2α (Fig 6B and 6C), total expression levels of CHOP (Fig 6B and 6D), and phosphorylation levels of JNK (Fig 6B and 6E) were markedly reduced. These results demonstrate that the daily administration of bromocriptine prevented hepatic ER stress in diet-induced obese mice.

### Preconditioning effects of bromocriptine to prevent severe ER stress in HepG2 cells

**Fig 7:**
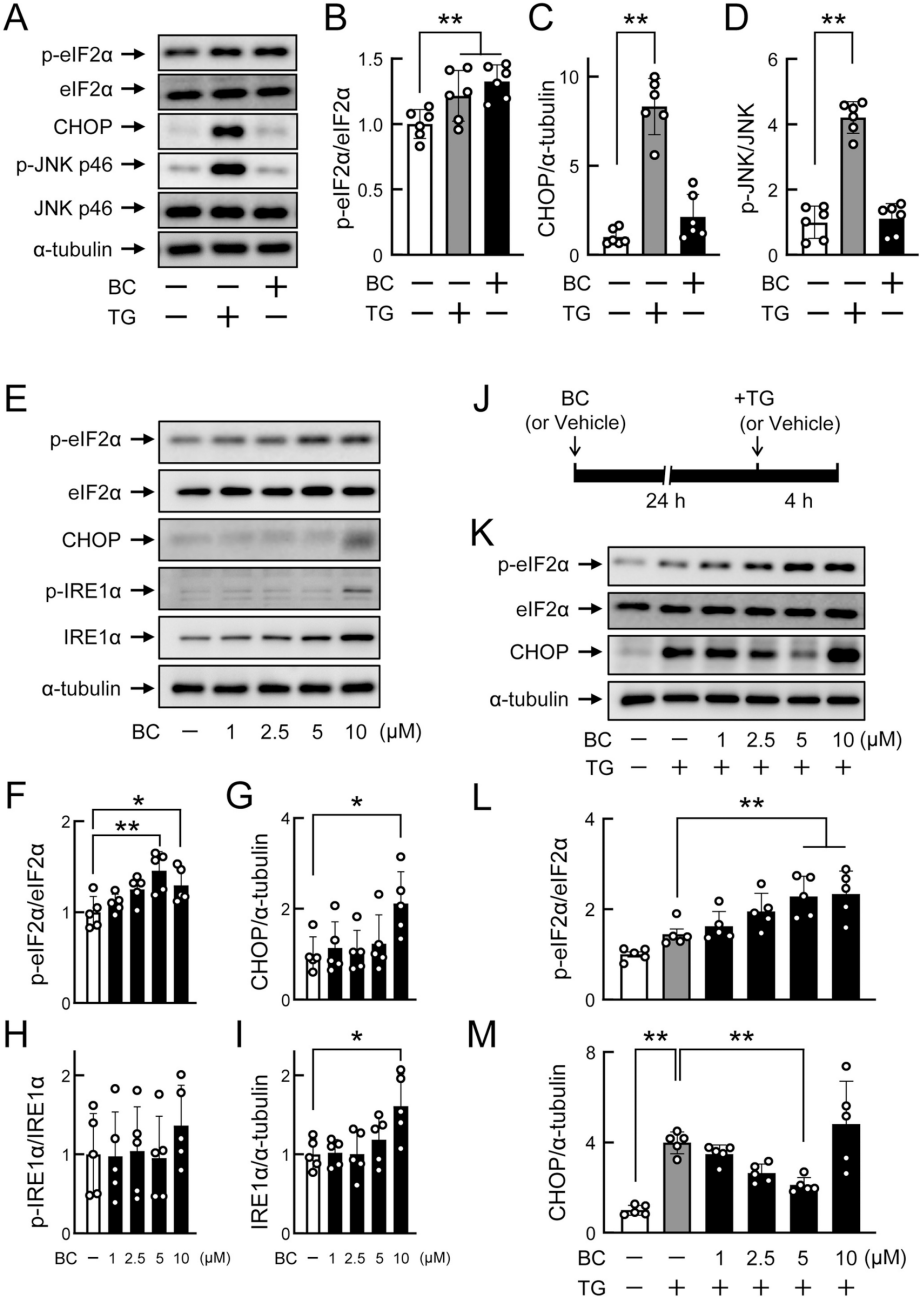
Direct effects of bromocriptine on ER stress levels in HepG2 cells in the presence and absence of thapsigargin. (A-D) Comparison of bromocriptine (BC)- and thapsigargin (TG)-induced ER stress in HepG2 cells. Cells seeded and incubated for 24 h were treated with vehicle (0.05% DMSO) for 28 h, BC (5 µM) for 28 h, or a vehicle pretreatment (for 24 h) plus TG (100 nM for 4 h). n = 6 per group. (A) Representative Western blot images. (B-D) Effects of BC and TG on the levels of p-eIF2α/eIF2α, CHOP/α-tubulin, and p-JNK/JNK. (E-I) Induction of mild ER stress by BC. HepG2 cells seeded and incubated for 24 h were treated with BC (1–10 µM) or vehicle for 24 h. n = 5 per group. (E) Representative Western blot images. (F-I) Effects of BC on the levels of p-eIF2α/eIF2α, CHOP/α-tubulin, p-IRE1α/IRE1α, and IRE1α/α-tubulin. (J-M) Preconditioning effects of BC to prevent TG-induced severe ER stress. (J) Timeline of experimental procedures. HepG2 cells seeded and incubated for 24 h were pretreated with BC (1–10 µM) or vehicle for 24 h, and then treated with TG (100 nM) or vehicle (0.05% DMSO) for 4 h. n = 5 per group. (K) Representative Western blot images. (L-M) Effects of BC on the levels of p-eIF2α/eIF2α and CHOP/α-tubulin in the presence of TG. Values are expressed as the means ±  S.D. * *p* < 0.05 and **p < 0.01 by a one-way ANOVA with Dunnett’s test.

To clarify the mechanisms underlying the preventive effects of bromocriptine against hepatic ER stress, we investigated the direct effects of bromocriptine in human hepatoma HepG2 cells. The treatment with bromocriptine (5–10 µM) for 24 h or thapsigargin (100 nM, a potent ER stress inducer) for 4 h did not affect cell viability detected using a standard trypan blue exclusion method. Bromocriptine (5 µM, 28 h) and thapsigargin (100 nM, 4 h) both increased the phosphorylation levels of eIF2α, a pan-marker of ER stress (Fig 7A and 7B). In contrast, the expression of CHOP and phosphorylation of JNK, both of which are markers of severe ER stress, were induced by thapsigargin (100 nM, 4 h), but not by bromocriptine (5 µM, 28 h) (Fig 7A, 7C and 7D), indicating that 5 µM bromocriptine and 100 nM thapsigargin caused mild and severe ER stress, respectively. When HepG2 cells were treated with bromocriptine (1–10 µM) for 24 h, eIF2α phosphorylation levels were increased by the drug at 5–10 µM (Fig 7E and 7F), whereas a higher concentration (10 µM) was needed to increase CHOP expression (Fig 7E and 7G). The up-regulated expression of IRE1α was also induced by bromocriptine at 10 µM, whereas its phosphorylation level remained unchanged (Fig 7E, 7H and 7I). Under such conditions, the PEPCK1 expression was not affected by bromocriptine (1–10 µM) (S3 Fig).

Since mild ER stress has been reported to prevent subsequent severe stress [19,20], we investigated whether bromocriptine exerted preventive effects against thapsigargin-induced severe ER stress in HepG2 cells. Cells were pretreated with bromocriptine (5 µM) for 24 h and then exposed to thapsigargin (100 nM) for 4 h (Fig 7J). The results obtained showed that thapsigargin-induced increases in the phosphorylation levels of eIF2α were enhanced by bromocriptine (5–10 µM) (Fig 7K and 7L). Moreover, thapsigargin-induced increases in CHOP expression were suppressed by bromocriptine (1–5 µM) in a concentration-dependent manner, while bromocriptine at 10 µM did not exert this effect (Fig 7K and 7M). Similarly, CPA (50 µM, another ER stressor)-induced phosphorylation of eIF2α was enhanced by bromocriptine (5–10 µM), whereas CPA (50 µM)-induced CHOP expression was suppressed by 5 µM but not 10 µM of bromocriptine (S4 Fig). Therefore, low concentrations of bromocriptine were beneficial for the prevention of severe ER stress in a hepatic cell line.

## Discussion

The present study demonstrated that bromocriptine elicited biphasic effects on glucose metabolism in mice. The single administration of bromocriptine induced an acute and transient increase in blood glucose levels, whereas its long-term daily administration improved glucose tolerance in diet-induced obese mice and genetically-obese *db/db* mice. Neither dopamine D2 receptors nor the ANS were involved in the beneficial long-term effects of bromocriptine, indicating the involvement of the peripheral effects of bromocriptine. The daily administration of bromocriptine mitigated ER stress in the livers of diet-induced obese mice. Furthermore, in cultured human hepatoma HepG2 cells, bromocriptine attenuated thapsigargin-induced ER stress. These results suggest that bromocriptine prevents the development of type 2 diabetes at least partly by promoting the maintenance of hepatic ER homeostasis.

Bromocriptine has been reported to acutely inhibit glucose-stimulated insulin secretion via α2-adrenergic receptor [7] or both α2-adrenergic and dopamine D2 receptors in pancreatic β-cells [15]. In addition, α2-adrenergic receptor agonists, such as clonidine and dexmedetomidine, were found to reduce serum insulin levels and increase blood glucose levels [40,41], and the effects of clonidine were mediated by the activation of spinal α2-adrenergic receptors and the sympathetic nervous system. In the present study, we showed that basal blood glucose levels were acutely elevated by bromocriptine (10 mg/kg) in mice, and this effect was diminished under prolonged fasting conditions in which circulating insulin levels decreased. We also observed that bromocriptine (10 mg/kg) tended to reduce serum insulin levels, consistently with a previous study [7]. The glucose-elevating effects of bromocriptine were not affected by a deficiency of dopamine D2 receptors, but were completely inhibited by yohimbine and partly by hexamethonium, indicating the requirement of α2-adrenergic receptors and the partial involvement of the ANS in the effects of bromocriptine. These results suggest that bromocriptine acutely induced hyperglycemia by inhibiting pancreatic insulin secretion through the activation of α2-adrenergic receptors. Additionally, some other α2-adrenoceptor-mediated mechanism in the spinal cord and/or the ANS might also contribute to its hyperglycemic effect.

Regarding the long-term effects of bromocriptine, its daily administration has been reported to ameliorate glucose metabolism in various animal models of metabolic disorders, including genetically obese *ob/ob* mice, diet-induced obese melanocortin-4 receptor knockout and prolactin knockout mice, and dogs fed HFD [8–11,13,42,43]. Among these studies, the difference in the vehicles used does not affect the effectiveness of bromocriptine. We consistently observed that the daily administration of bromocriptine improved glucose tolerance in mice fed HFD and genetically obese *db/db* mice. Bromocriptine is a well-known dopamine D2 receptor agonist, and a close relationship has been reported between the dopamine D2 receptor system and the regulation of glucose homeostasis [5]. However, bromocriptine-induced improvements in glucose tolerance were observed in dopamine D2 receptor knockout mice fed HFD. To the best of our knowledge, this is the first study to show that bromocriptine was metabolically beneficial independently of dopamine D2 receptors under type 2 diabetic conditions.

The brain plays a crucial role in the maintenance of metabolic homeostasis [44]. The beneficial effects of the circadian-timed administration of bromocriptine on glucose metabolism have been attributed to its central effects, which regulate the central biological clock system and reduce sympathetic tone [4]. In addition, a systemic injection of bromocriptine has been reported to induce biphasic effects on sleep and wakefulness, such that it drives arousal at high doses, but also promotes slow-wave sleep and rapid eye movement sleep at low doses [21]. However, a recent study demonstrated that circadian disruption and changes in the timing of treatment did not affect bromocriptine-induced improvements in glucose tolerance [13]. The present study indicated that the central effects of orexin and melatonin, which coordinate the link between the sleep-wake cycle and glucose metabolism [45], were not required for the beneficial effects of bromocriptine. More importantly, we found that a pretreatment with the autonomic ganglion blocker hexamethonium did not have an impact on the effects of the daily bromocriptine administration on glucose intolerance in diet-induced obese mice. These results suggest that the site of the metabolically beneficial effects of bromocriptine is located downstream of the ANS, i.e., in peripheral tissues.

In terms of its peripheral effects, it has been considered that bromocriptine regulates glucose metabolism in a tissue-specific manner. For instance, a previous *in vivo* study has shown that 2-week daily administration of bromocriptine improved insulin signaling in the skeletal muscle of type 2 diabetic rats [46]. In dogs fed HFD, circadian-timed administration of bromocriptine for 4 weeks improved glucose disposal particularly in the skeletal muscle during a hyperinsulinemic hyperglycemic clamp [43]. An *ex vivo* study [14] has shown that bromocriptine enhanced insulin-induced glucose uptake in the rat mesenteric white adipose tissue. In contrast, it increased glucose uptake in the liver tissue in an insulin-independent manner. The mechanism of how bromocriptine promotes hepatic glucose metabolism has remained unclear. In the obese and type 2 diabetic states, chronic ER stress is a major cause of increased hepatic glucose production that leads to hyperglycemia and glucose intolerance [17,47,48]. Bromocriptine was shown to prevent the leakage of ER resident proteins (referred to as ‘ER exodosis’) during the stimulation of cultured human skeletal muscle cells and neuronal cells with thapsigargin, an ER stress inducer [10]. The beneficial effect of bromocriptine on ER homeostasis was independent of dopamine D2 receptors. In the present *in vivo* study, we found that the chronic effect of bromocriptine was associated with changes in hepatic ER stress response. In the absence of bromocriptine, marked eIF2α phosphorylation and CHOP expression were observed in the livers of diet-induced obese mice. These abnormalities are consistent with the characteristics of chronic ER stress, in which protein kinase RNA-like ER kinase (PERK)-eIF2α-CHOP signaling is continuously activated [49]. The daily administration of bromocriptine reduced the levels of the phosphorylated eIF2α and total CHOP expression, indicating that bromocriptine mitigated chronic hepatic ER stress. Since sustained ER stress sufficiently promotes hepatic glucose output and hyperglycemia [17], bromocriptine appears to prevent obesity-induced glucose intolerance at least partly by reducing hepatic ER stress.

Short-term mild ER stress is known to exert hormetic effect (i.e., preconditioning) against long-term severe ER stress [19,20,34,38]. In our *in vitro* experiment, bromocriptine induced bidirectional effects on hepatic ER stress depending on the concentrations used. High concentrations of bromocriptine increased both eIF2α phosphorylation and CHOP expression levels in HepG2 cells, similar to thapsigargin, whereas a low concentration of bromocriptine increased phosphorylated eIF2α levels, but not CHOP expression levels. The transient and prolonged expression of the CHOP protein are regarded as indices of mild (i.e., adaptive) and severe (i.e., lethal) ER stress, respectively [19]. Importantly, the pretreatment with bromocriptine at a low concentration (5 μM) attenuated thapsigargin-induced severe ER stress responses. Therefore, bromocriptine is considered to be a drug to induce beneficial ‘mild’ ER stress that promotes preconditioning to prevent ‘severe’ hepatic ER stress. Bromocriptine caused such modulations of ER stress without affecting the gluconeogenic PEPCK1 expression in HepG2 cells, suggesting that hepatic ER stress, but not hepatic glucose production, may be the primary target of bromocriptine.

Bromocriptine is metabolized through the same principal pathway in mice, rat, dog, monkey, and humans [50]. After administration, circulating concentrations of bromocriptine vary over a wide range in a time-dependent manner, i.e., peak concentrations that are reached 1–3 h after oral administration are followed by a rapid decrease with a half-life of 3–7 h in humans [51]. Similar pharmacokinetics has been reported in mice injected intravenously with bromocriptine-loaded nanoparticles [52]. It has been reported that bromocriptine at a high dose (6 mg/kg) and at a low dose (2 mg/kg) promotes wakefulness and sleep, respectively, in rats, indicating that dosage differences of up to three-fold can induce the opposite effect [21]. Therefore, it is difficult to assume the relationship between the *in vivo* and *in vitro* efficacy of bromocriptine; however, a previous study has reported that bromocriptine (10 µM and 10 mg/kg, i.p.) stimulates the same antioxidative mechanisms *in vitro* and *in vivo* [53]. Similarly, we observed that bromocriptine (10 µM and 10 mg/kg, i.p.) elicited the CHOP expression *in vitro* (Fig 7G and 7M) and *in vivo* (Fig 2J), whereas bromocriptine (5 µM) caused the opposite effect *in vitro* (Fig 7M). Based on these profiles, we suppose that bromocriptine (10 mg/kg) might produce the same effect *in vivo* as it did at 10 µM *in vitro*, but intense hepatic ER stress elicited by high concentrations of circulating bromocriptine shortly after the administration may switch to the opposite effect rapidly within a few hours, equivalent to its half-life. We also noticed that there is a slight discrepancy between bromocriptine’s effects on eIF2α phosphorylation *in vivo* (i.e., decrease, Fig 6) and *in vitro* experiments (i.e., increase, Fig 7). Since short-term repeated stress exposure changes physiological functions to achieve homeostasis [54], periodical bromocriptine’s stimuli might activate some adaptive mechanism, thereby gradually promoting habituation to ER stress and improving glucose tolerance. Further study is needed to clarify the mechanism underlying the fine tuning of ER homeostasis by bromocriptine.

There are some limitations that need to be addressed. We showed that dopamine D2 and D1 receptors, α2-adrenergic receptors, ganglionic nicotinic acetylcholine receptors, leptin receptors, orexin, and melatonin receptors were not involved in the beneficial effects of the daily administration of bromocriptine on glucose metabolism; however, we did not identify the specific receptor responsible for the effects of this drug. Since bromocriptine binds to several monoamine receptors, multiple intracellular signaling pathways in peripheral tissues may be involved in the beneficial effects of bromocriptine. Indeed, some studies suggested that bromocriptine does not have a specific receptor to improve glucose metabolism [1]. These complex mechanisms need to be clarified in the future. Furthermore, we did not examine the effects of the QR formulation of bromocriptine (i.e., bromocriptine-QR used as anti-diabetic drug in the USA) in our *in vivo* and *in vitro* experiments; therefore, we cannot completely exclude the possibility that bromocriptine with different pharmacokinetics/pharmacodynamics profiles may have different effects under type 2 diabetic conditions.

## Conclusions

The present study provides the evidence to show that bromocriptine improved obesity-induced glucose intolerance in a dopamine D2 receptor-independent manner. In this process, bromocriptine appears to promote preconditioning to prevent severe hepatic ER stress induced by nutrient overload under obese type 2 diabetic conditions. These unique mechanisms may underlie the synergistic enhancement of anti-diabetic effects by bromocriptine and commonly used medicines [4]. Therefore, further research on chronotherapy using ER stress relievers, such as bromocriptine, is warranted to improve treatment for type 2 diabetes.

## Supporting information

S1 Fig No effect of bromocriptine on the total 24-h food intake in C57BL/6J mice. Male C57BL/6J mice (9 weeks old) fasted for 24 h were administered bromocriptine (BC, 10 mg/kg, i.p.) or vehicle (10% ethanol), and then refed for 24 h. Total amount of food ingested during 24 h refeeding was measured. n = 6 per group. Values are expressed as the means ±  S.D.
(TIF)

S2 Fig Acute effect of bromocriptine on serum insulin levels in C57BL/6J mice. Male C57BL/6J mice (9 weeks old) fed NCD ad libitum were administered bromocriptine (10 mg/kg, i.p.) or vehicle (10% ethanol). Food was removed just after the drug administration. Serum insulin levels were measured at indicated time points. n = 8 per group. Values are expressed as the means ±  S.D. The p-values were determined by the Student’s t-test.
(TIF)

S3 Fig No effect of bromocriptine on the gluconeogenic PEPCK1 expression in HepG2 cells. HepG2 cells seeded and incubated for 24 h were treated with bromocriptine (BC, 1-10 µM) or vehicle for 24 h. (A) Representative Western blot images for panel B. (B) Effects of BC on the levels of PEPCK1/α-tubulin. n = 5 per group.
(TIF)

S4 Fig Effects of bromocriptine on CPA-induced ER stress in HepG2 cells. (A-D) Preconditioning effects of bromocriptine (BC) to prevent cyclopiazonic acid (CPA)-induced severe ER stress. (A) Timeline of experimental procedures. HepG2 cells seeded and incubated for 24 h were pretreated with BC (5 and 10 µM) or vehicle for 24 h, and then treated with CPA (50 µM) or vehicle (0.05% DMSO) for 4 h. n = 8 per group. (B) Representative Western blot images. (C-D) Effects of BC on the levels of p-eIF2α/eIF2α (C) and CHOP/β-actin (D). Values are expressed as the means ±  S.D. * p < 0.05 and **p < 0.01 by a one-way ANOVA with Dunnett’s test.
(TIF)

S1 Data Datasets. (XLSX)

S1 File Original images for blots and gels. (PDF)

## Abbreviations:

ANOVA: analysis of variance
ANS: autonomic nervous system
ATR: atropine
BC: bromocriptine
CNS: central nervous system
CHOP: C/EBP homologous protein
CPA: cyclopiazonic acid
eIF2α: eukaryotic initiation factor-2α
DMSO: dimethyl sulfoxide
ER: endoplasmic reticulum
*G6Pase*: glucose-6-phosphatase
HEX: hexamethonium
HFD: high-fat diet
IRE1α: inositol-requiring enzyme-1α
JNK: c-Jun N-terminal kinase
NCD: normal chow diet
*Orexin* ^*-/-*^: orexin knockout
PCR: polymerase chain reaction
*Pepck*: phosphoenolpyruvate carboxykinase
PERK: protein kinase RNA-like endoplasmic reticulum kinase
POB: phenoxybenzamine
PRO: propranolol
PZS: prazosin
QR: Quick release
RT: reverse transcription
S.D.: standard deviation
TG: thapsigargin
YHB: yohimbine
ZT: zeitgeber time
