# Supplementary figures and images for: Bromocriptine improves glucose tolerance in obese mice via central dopamine D2 receptor-independent mechanism
Source: PLoS One. 2025 Mar 26;20(3):e0320157. doi: 10.1371/journal.pone.0320157 (PMC11940610; doi:10.1371/journal.pone.0320157)

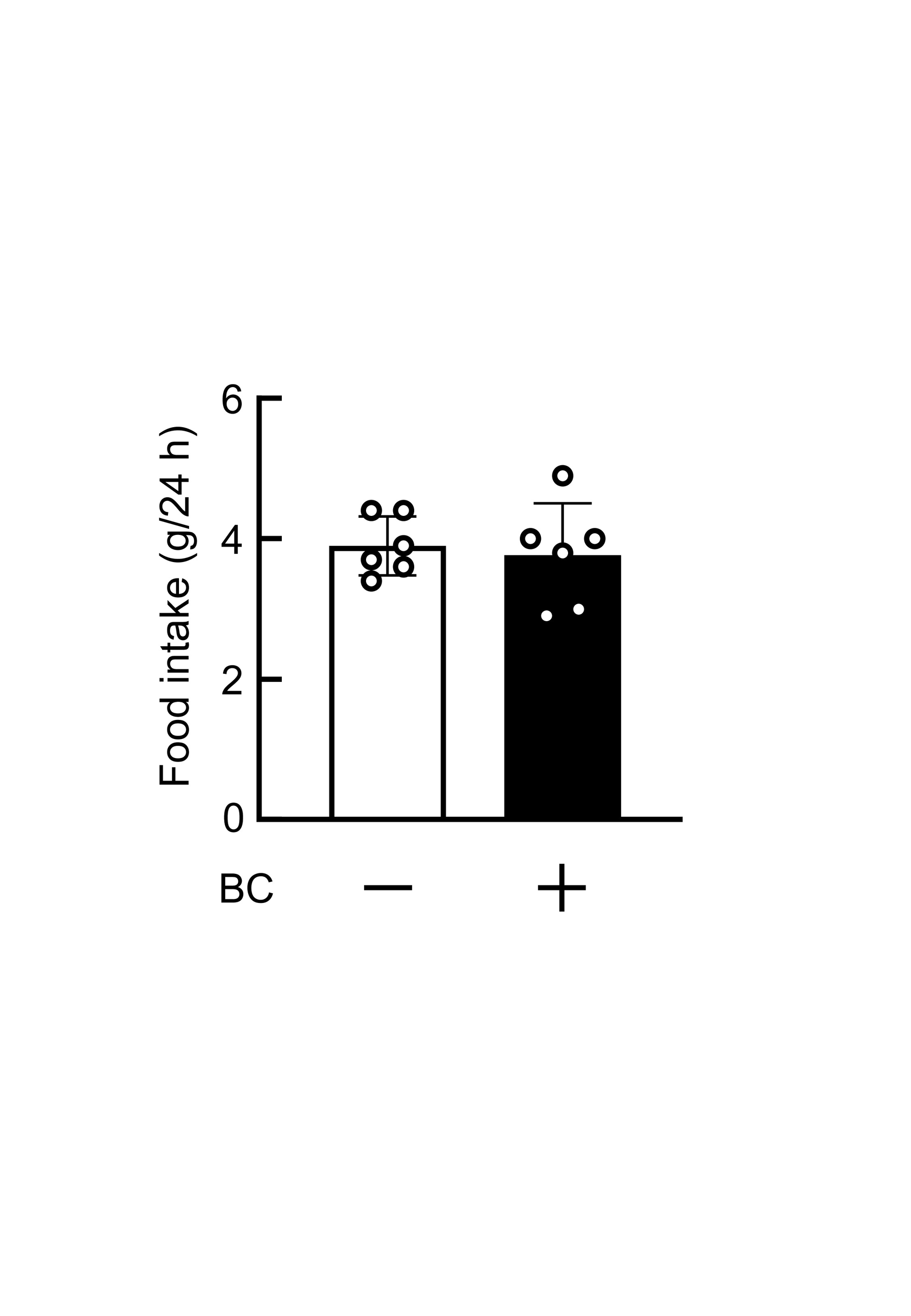

Supplement: S1 Fig — No effect of bromocriptine on the total 24-h food intake in C57BL/6J mice. Male C57BL/6J mice (9 weeks old) fasted for 24 h were administered bromocriptine (BC, 10 mg/kg, i.p.) or vehicle (10% ethanol), and then refed for 24 h. Total amount of food ingested during 24 h refeeding was measured. n = 6 per group. Values are expressed as the means ± S.D. (TIF) [file pone.0320157.s001.tif]

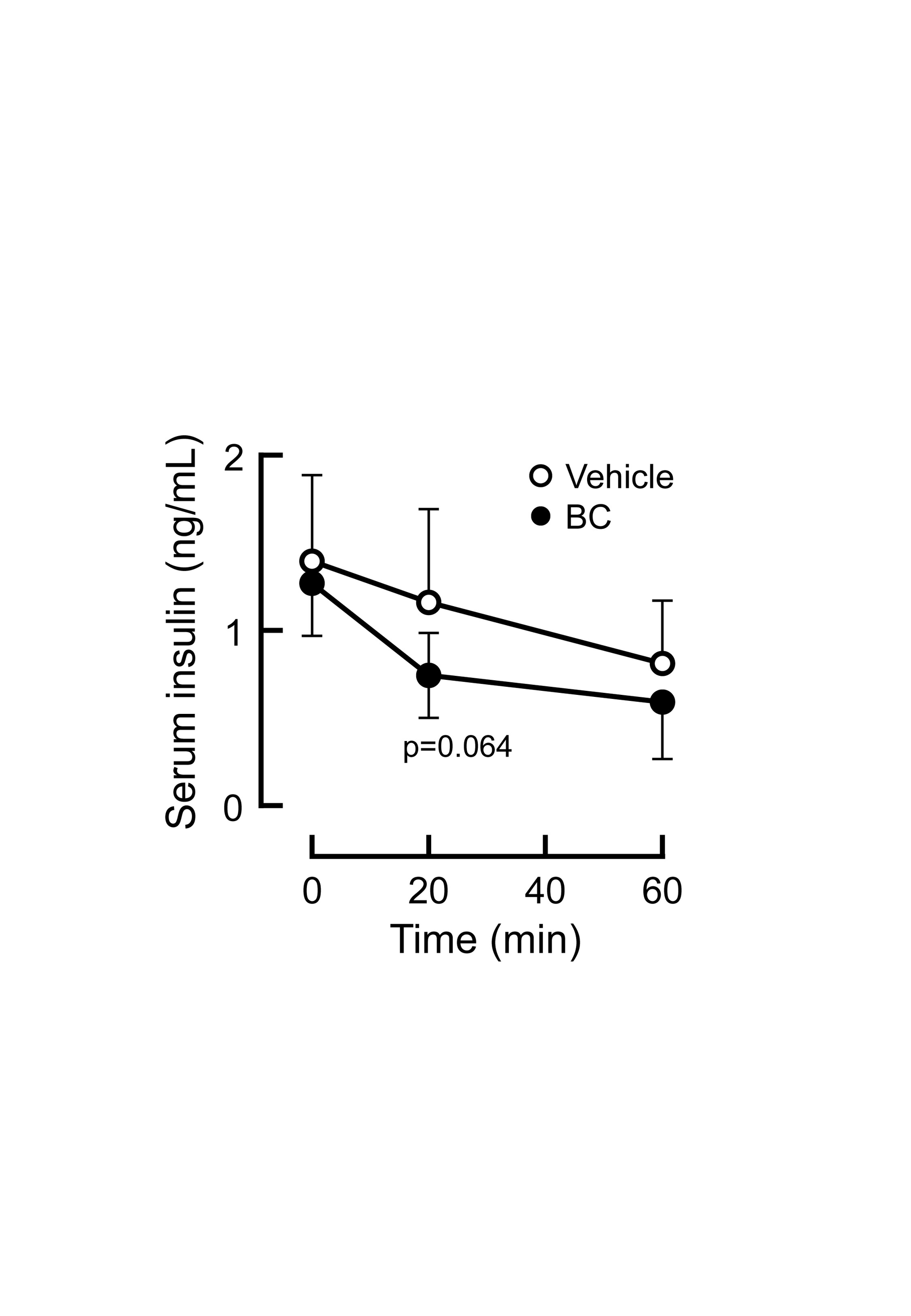

Supplement: S2 Fig — Acute effect of bromocriptine on serum insulin levels in C57BL/6J mice. Male C57BL/6J mice (9 weeks old) fed NCD ad libitum were administered bromocriptine (10 mg/kg, i.p.) or vehicle (10% ethanol). Food was removed just after the drug administration. Serum insulin levels were measured at indicated time points. n = 8 per group. Values are expressed as the means ± S.D. The p-values were determined by the Student’s t-test. (TIF) [file pone.0320157.s002.tif]

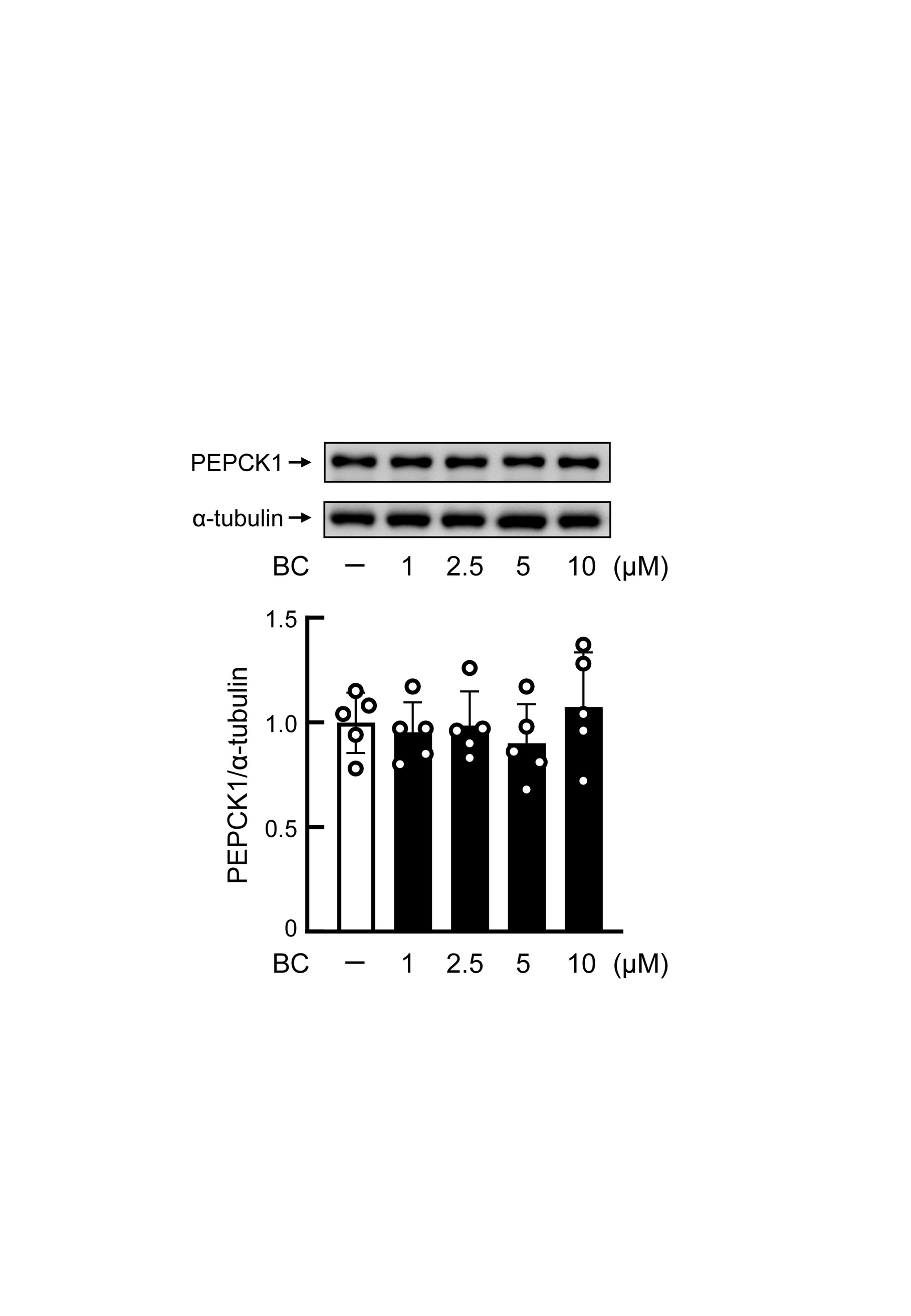

Supplement: S3 Fig — No effect of bromocriptine on the gluconeogenic PEPCK1 expression in HepG2 cells. HepG2 cells seeded and incubated for 24 h were treated with bromocriptine (BC, 1-10 µM) or vehicle for 24 h. (A) Representative Western blot images for panel B. (B) Effects of BC on the levels of PEPCK1/α-tubulin. n = 5 per group. (TIF) [file pone.0320157.s003.tif]

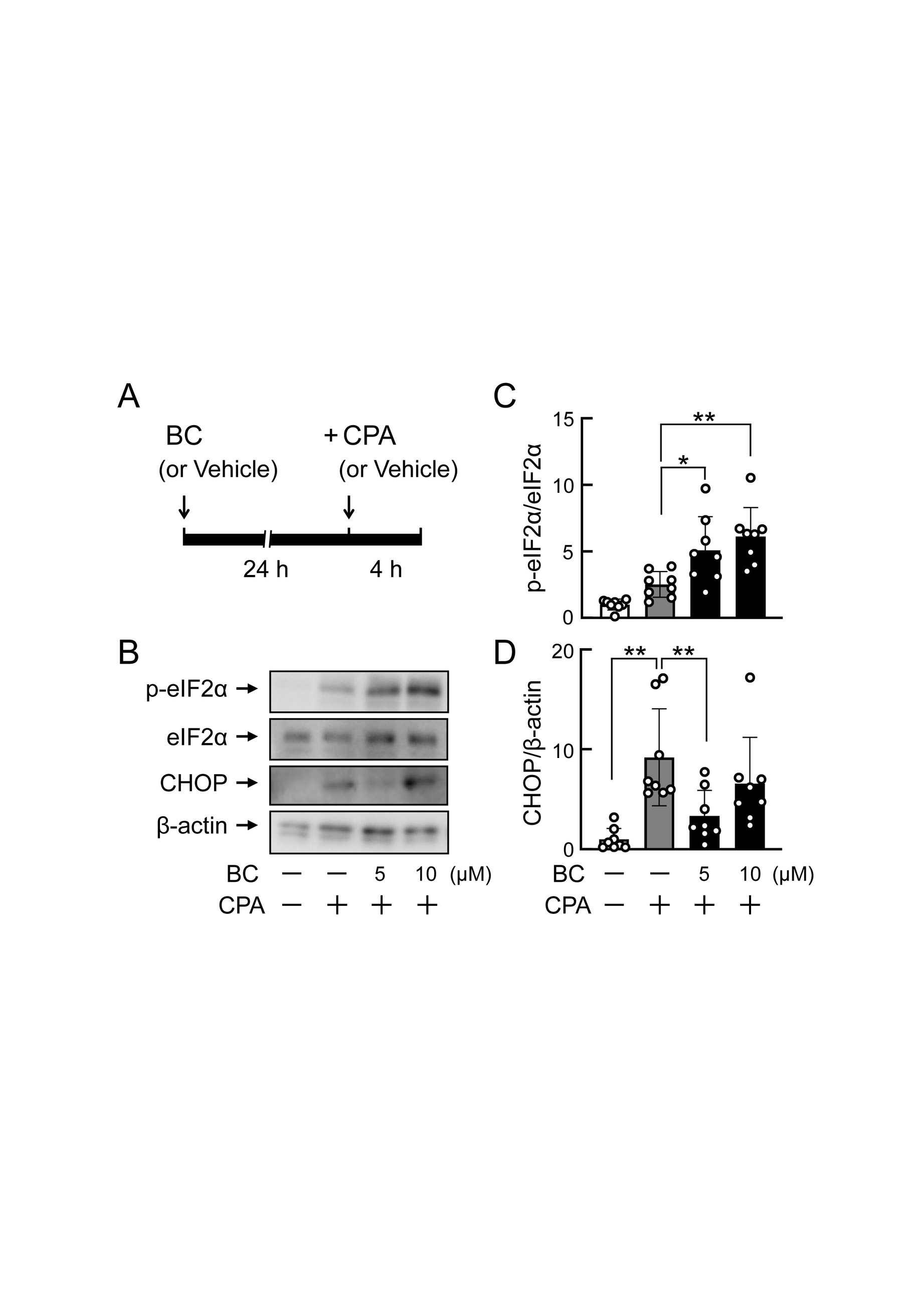

Supplement: S4 Fig — Effects of bromocriptine on CPA-induced ER stress in HepG2 cells. (A-D) Preconditioning effects of bromocriptine (BC) to prevent cyclopiazonic acid (CPA)-induced severe ER stress. (A) Timeline of experimental procedures. HepG2 cells seeded and incubated for 24 h were pretreated with BC (5 and 10 µM) or vehicle for 24 h, and then treated with CPA (50 µM) or vehicle (0.05% DMSO) for 4 h. n = 8 per group. (B) Representative Western blot images. (C-D) Effects of BC on the levels of p-eIF2α/eIF2α (C) and CHOP/β-actin (D). Values are expressed as the means ± S.D. * p < 0.05 and **p < 0.01 by a one-way ANOVA with Dunnett’s test. (TIF) [file pone.0320157.s004.tif]
