# Supplementary material for: Bromocriptine improves glucose tolerance in obese mice via central dopamine D2 receptor-independent mechanism
Source: PLoS One. 2025 Mar 26;20(3):e0320157. doi: 10.1371/journal.pone.0320157 (PMC11940610; doi:10.1371/journal.pone.0320157)
Supplement: S1 File — (PDF) [file pone.0320157.s006.pdf]

Figure 6B: Western-blot raw data

Immunoblots

Molecular weight markers on the same gel

p-eIF2α

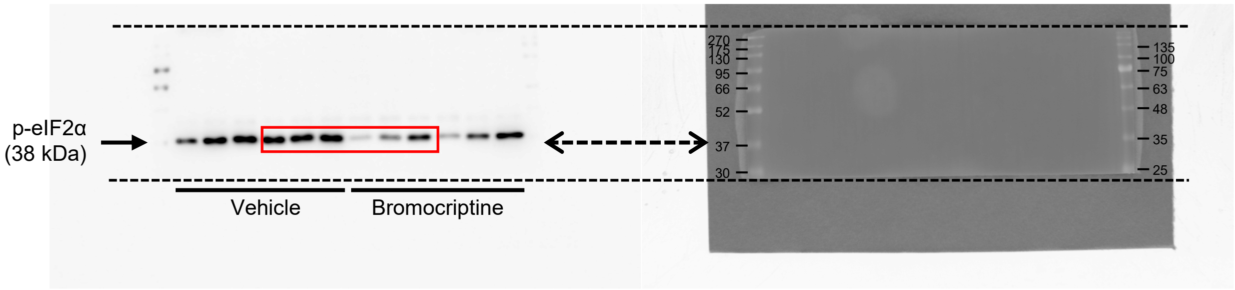

eIF2α

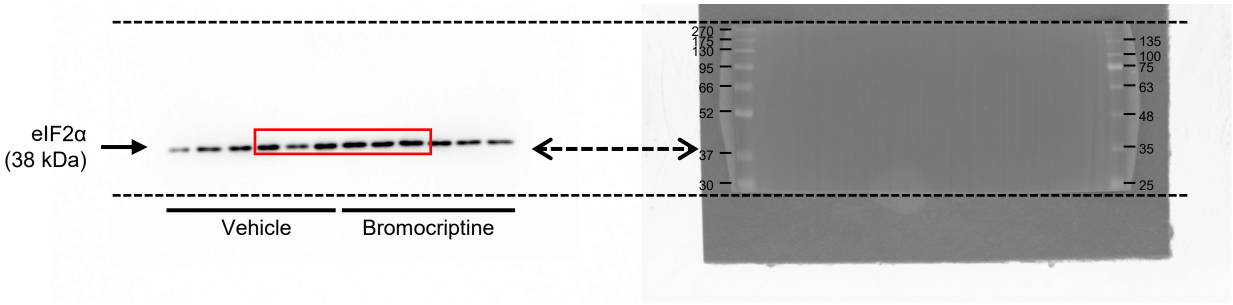

CHOP

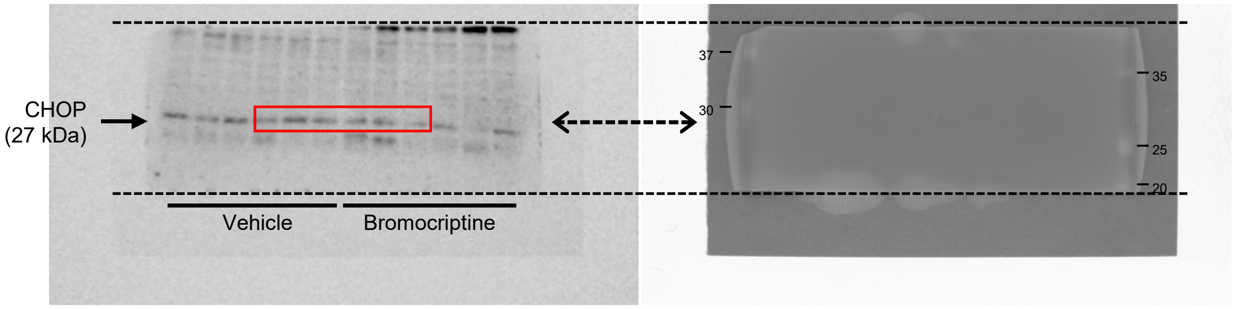

Capture method:
ImageQuant LAS4000

Figure 6B: Western-blot raw data

Immunoblots

Molecular weight markers on the same gel

p-JNK

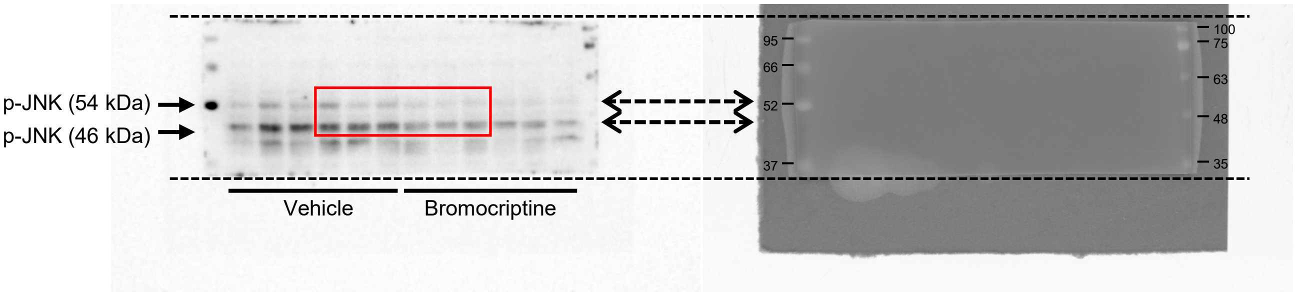

JNK

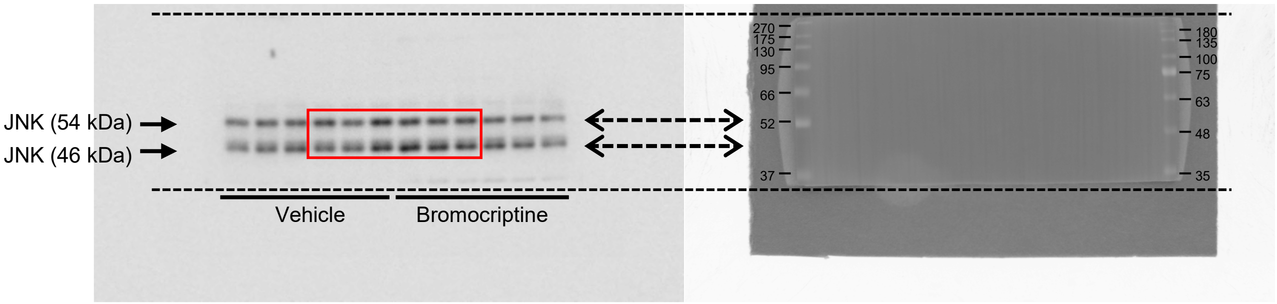

α-tubulin

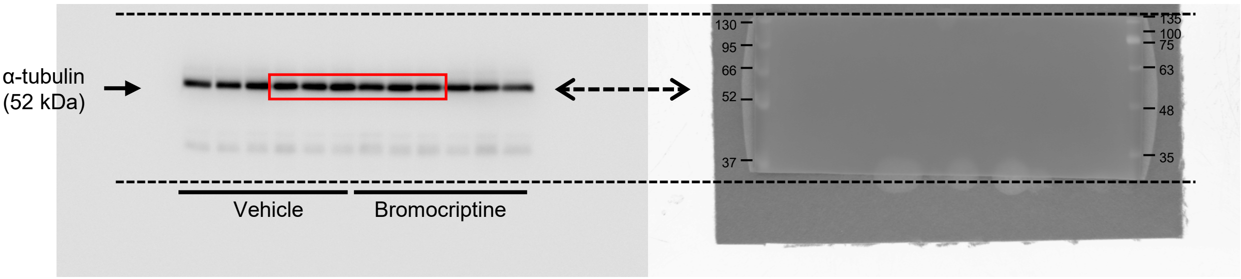

Capture method:
ImageQuant LAS4000

Figure 7A: Western-blot raw data

Immunoblots | Molecular weight markers on the same gel

p-eIF2α Repeat-1

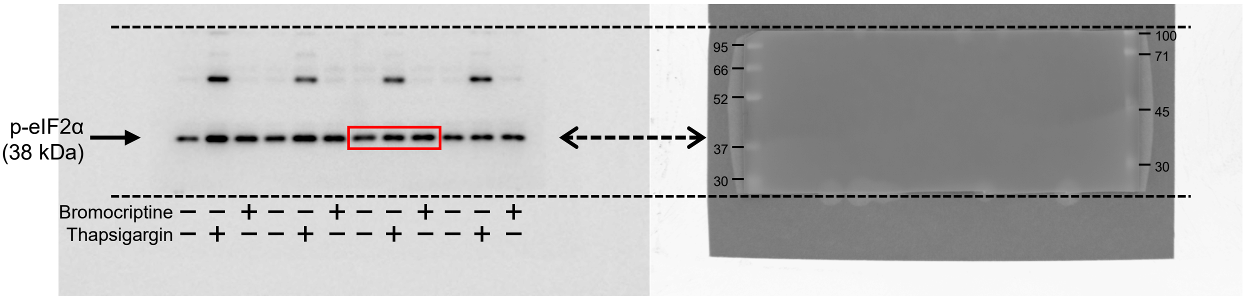

p-eIF2α Repeat-2

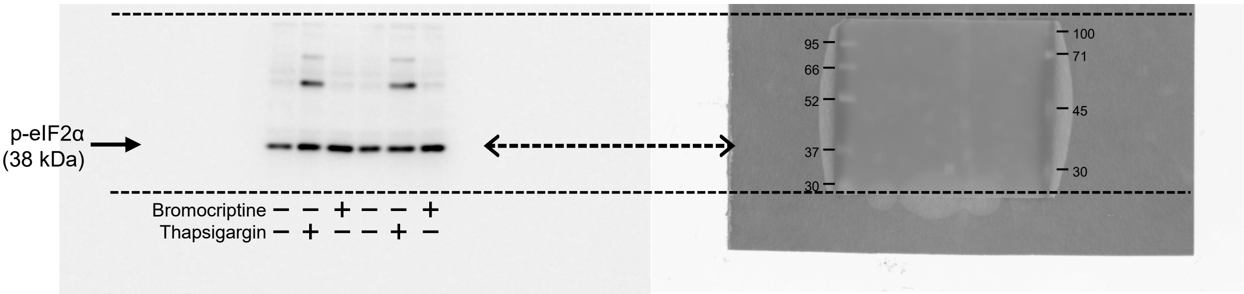

eIF2α Repeat-1

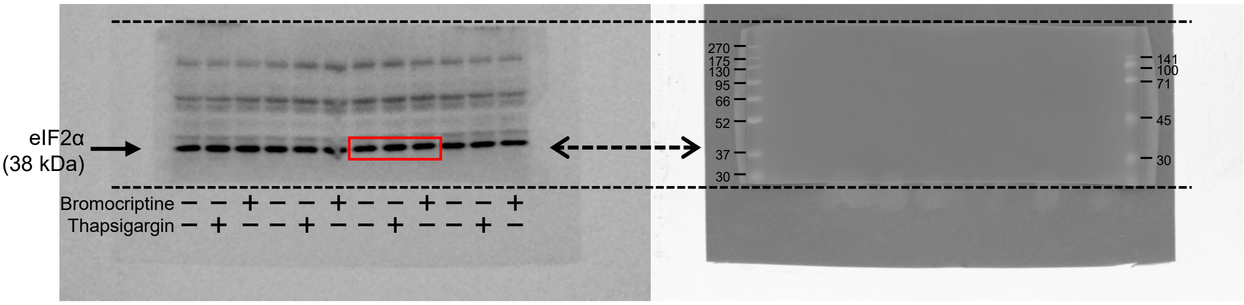

eIF2α Repeat-2

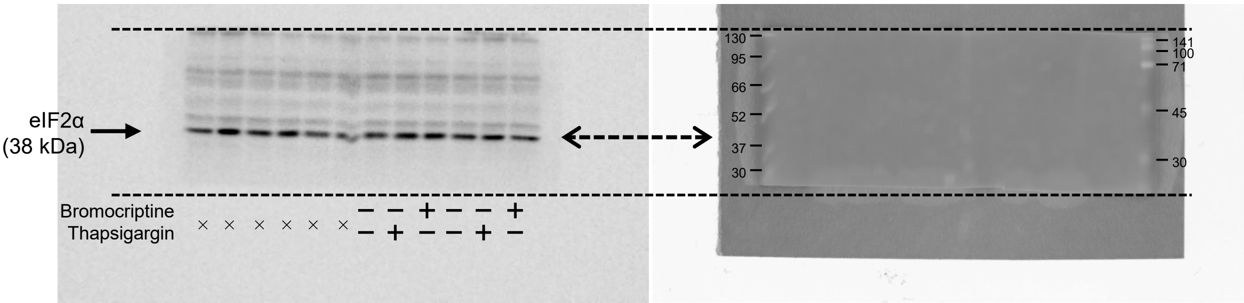

Capture method: ImageQuant LAS4000

Figure 7A: Western-blot raw data

Immunoblots

Molecular weight markers on the same gel

CHOP Repeat-1

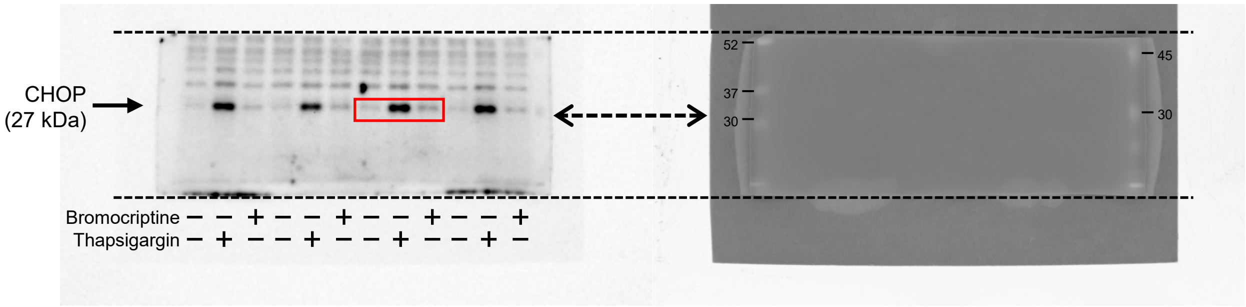

CHOP Repeat-2

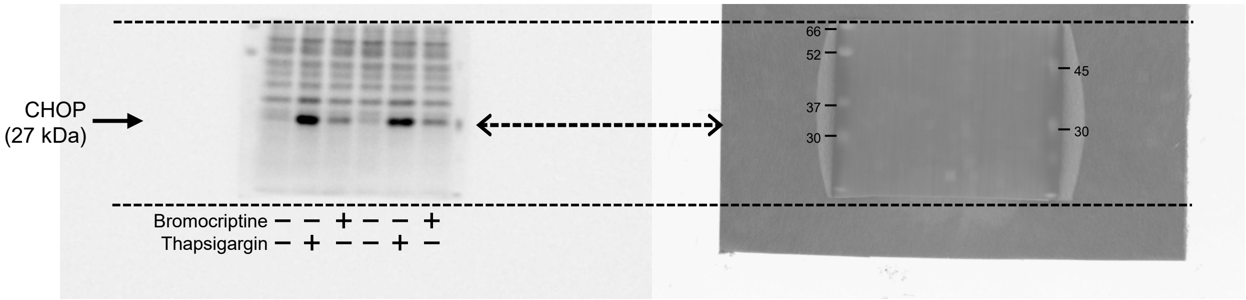

Capture method: ImageQuant LAS4000

Figure 7A: Western-blot raw data

Immunoblots | Molecular weight markers on the same gel

p-JNK Repeat-1

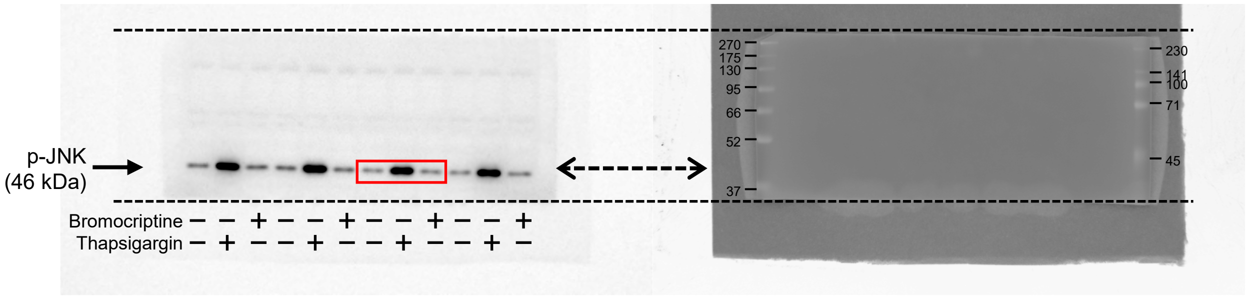

p-JNK Repeat-2

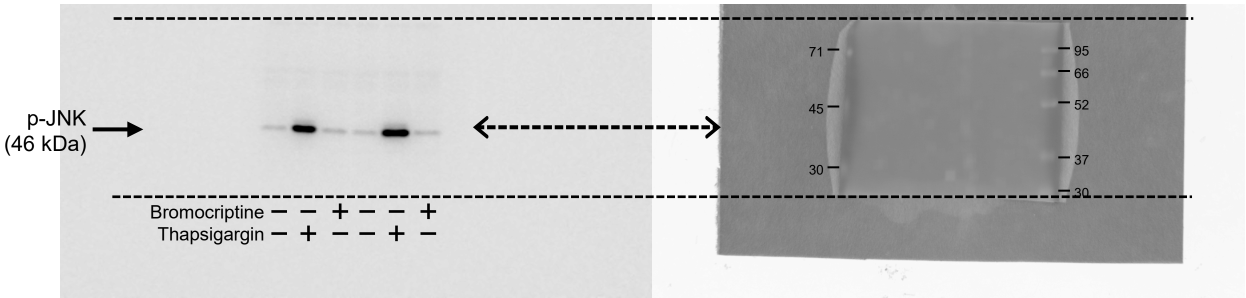

JNK Repeat-1

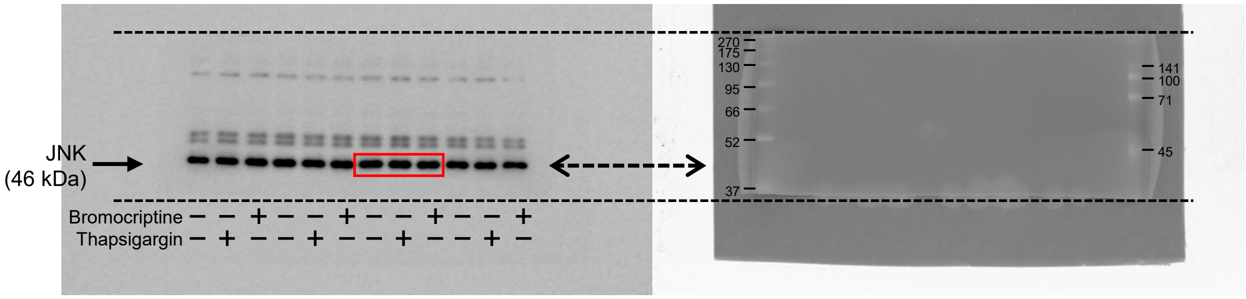

JNK Repeat-2

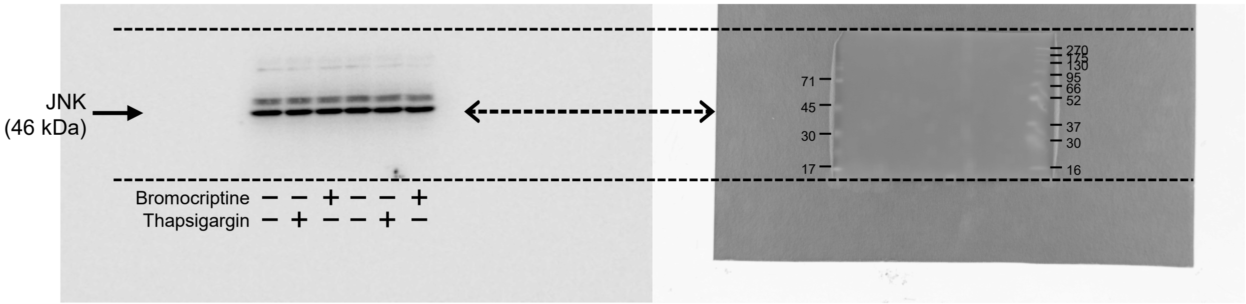

Capture method: ImageQuant LAS4000

Figure 7A: Western-blot raw data

Immunoblots

Molecular weight markers on the same gel

α-tubulin Repeat-1

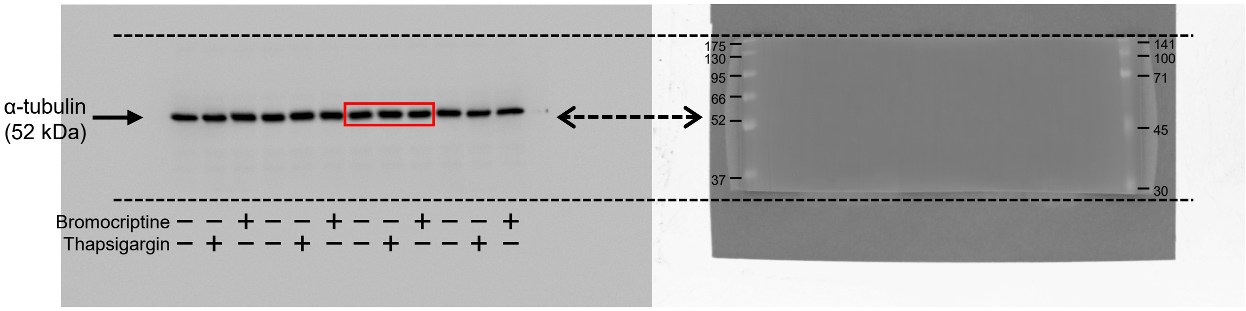

α-tubulin Repeat-2

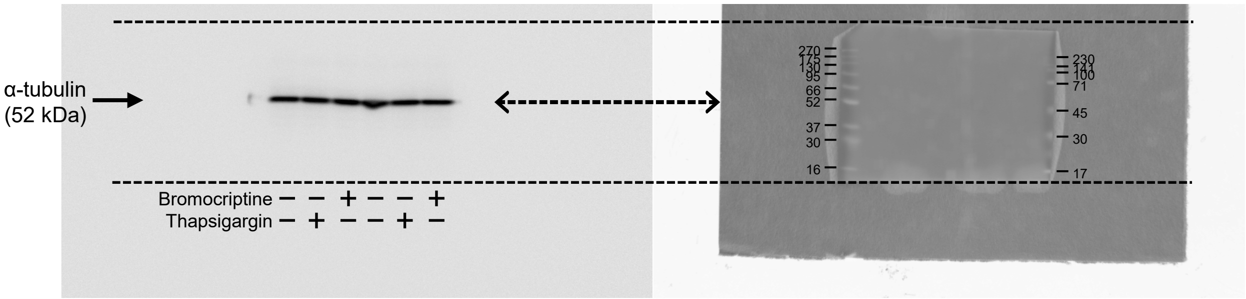

Capture method: ImageQuant LAS4000

Figure 7E: Western-blot raw data

Immunoblots

Molecular weight markers on the same gel

p-eIF2α Repeat-1

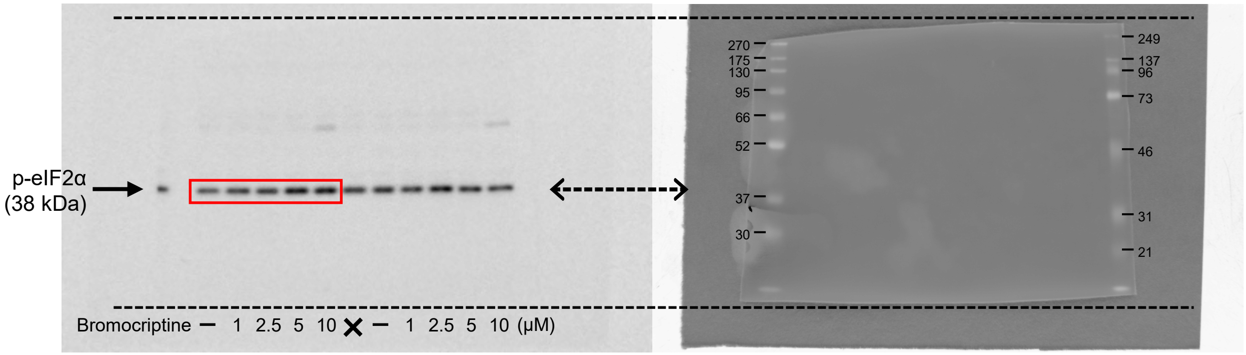

p-eIF2α Repeat-2

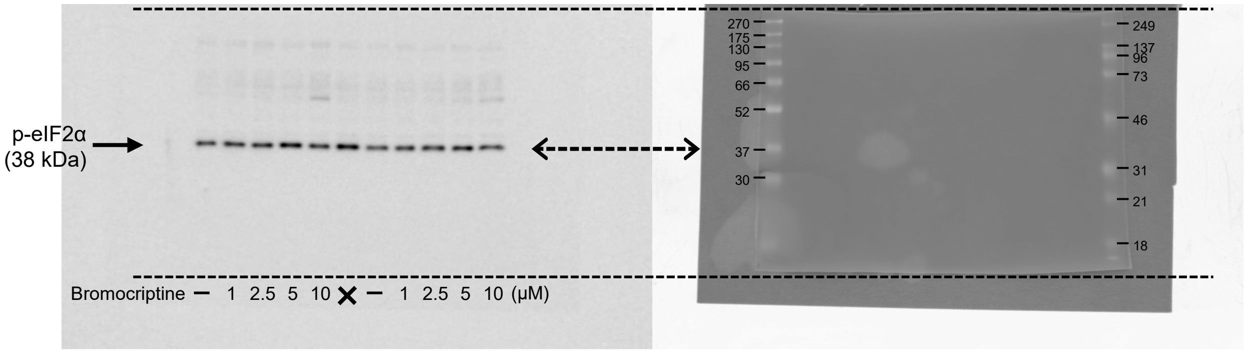

p-eIF2α Repeat-3

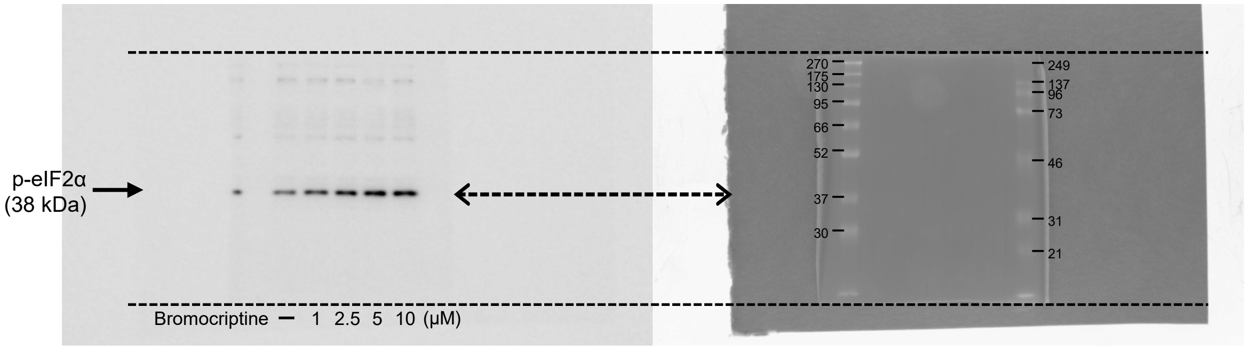

Capture method: ImageQuant LAS4000

Figure 7E: Western-blot raw data

Immunoblots | Molecular weight markers on the same gel

eIF2α Repeat-1

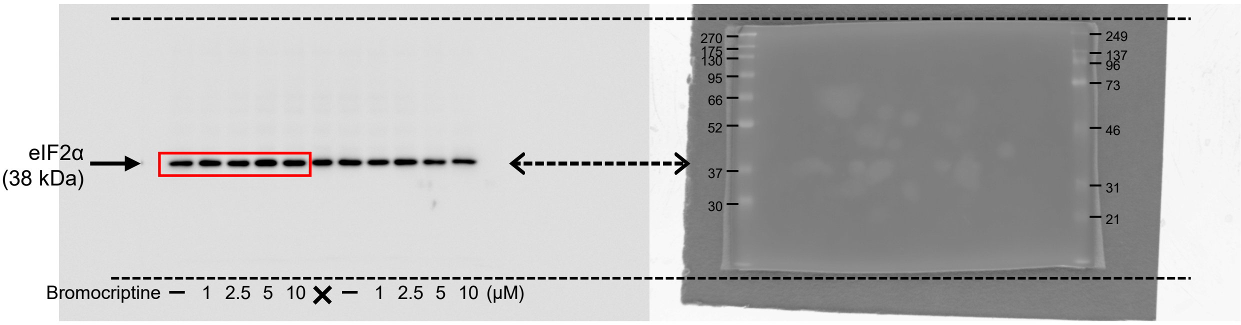

eIF2α Repeat-2

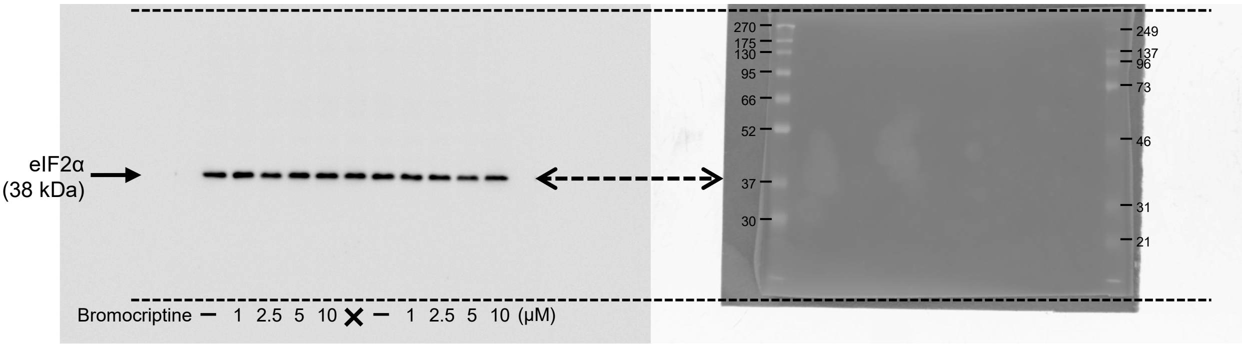

eIF2α Repeat-3

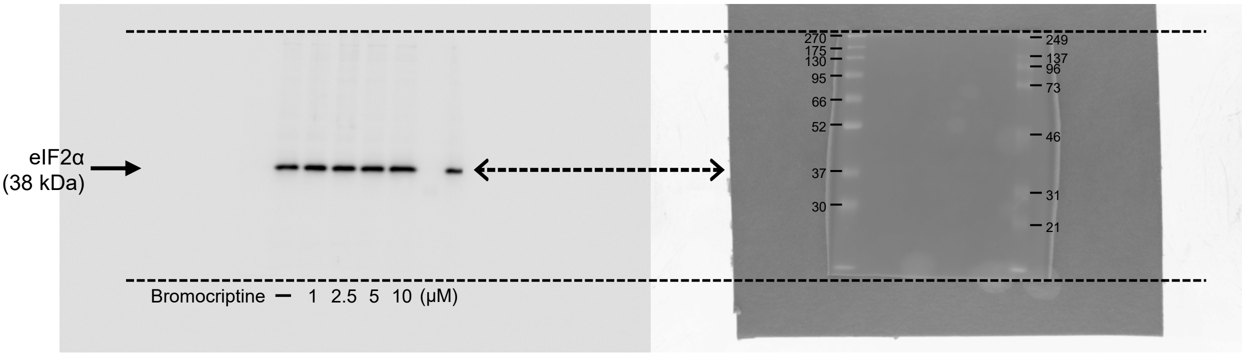

Capture method: ImageQuant LAS4000

Figure 7E: Western-blot raw data

Immunoblots | Molecular weight markers on the same gel

CHOP Repeat-1

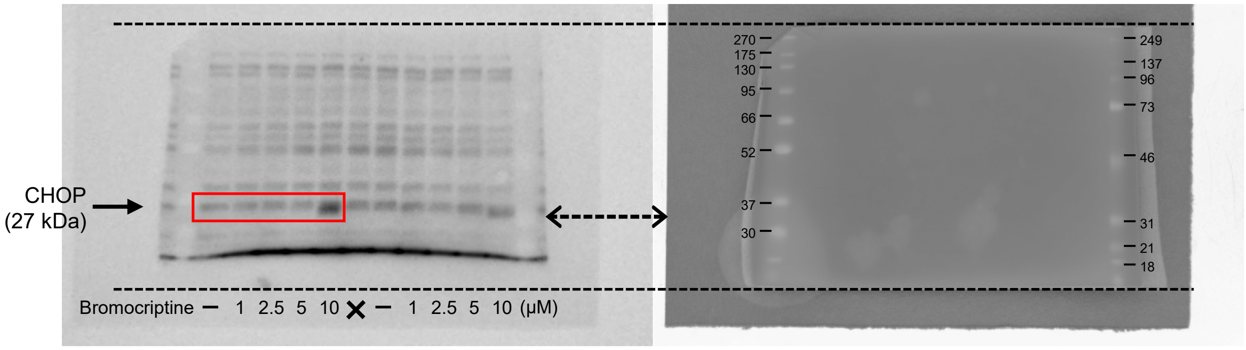

CHOP Repeat-2

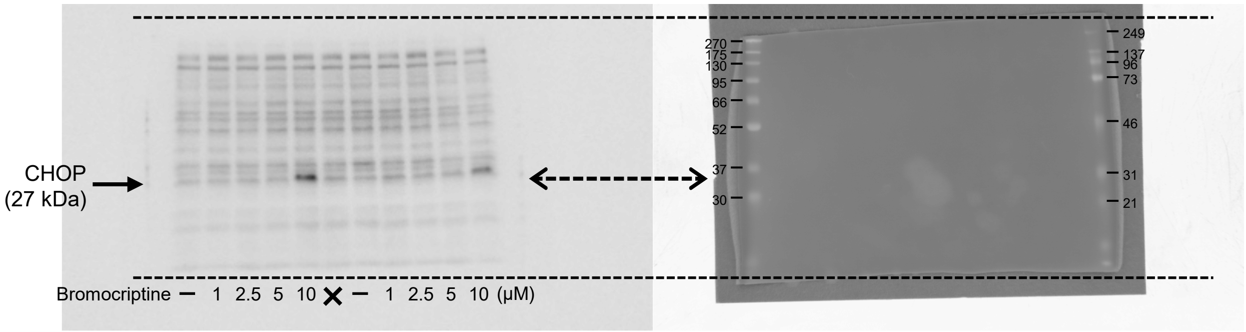

CHOP Repeat-3

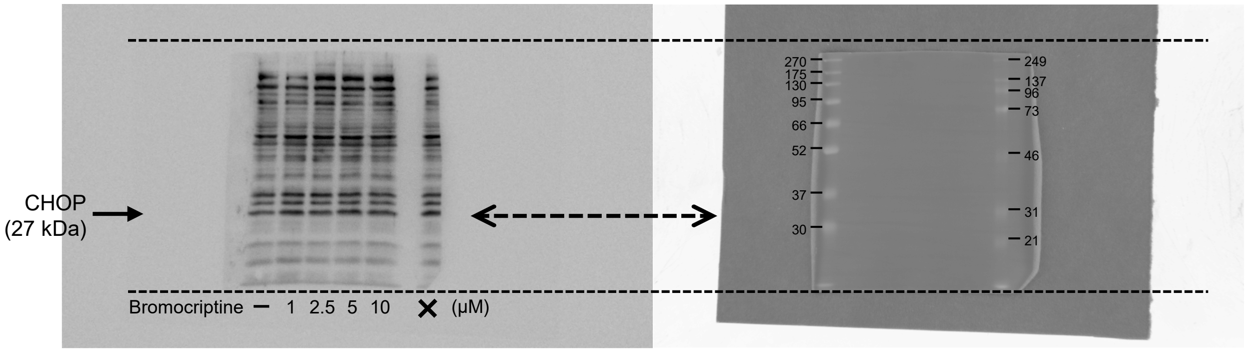

Capture method: ImageQuant LAS4000

Figure 7E: Western-blot raw data

Immunoblots | Molecular weight markers on the same gel

p-IRE1α Repeat-1

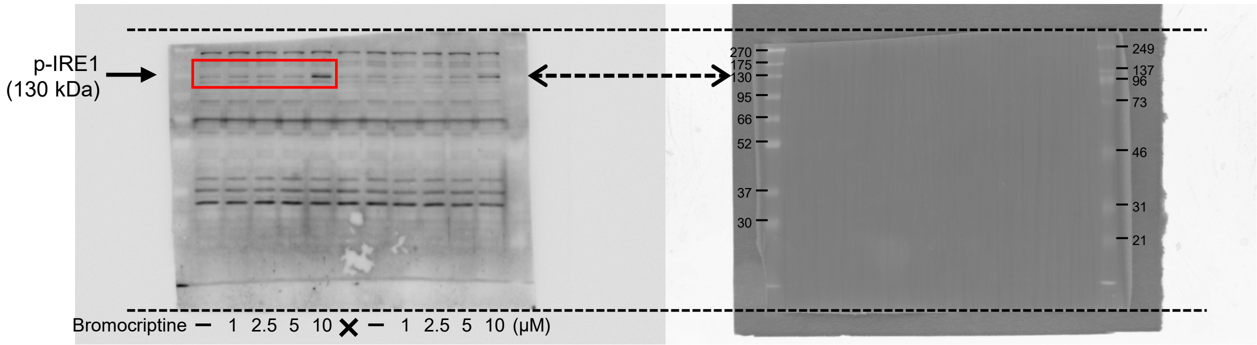

p-IRE1α Repeat-2

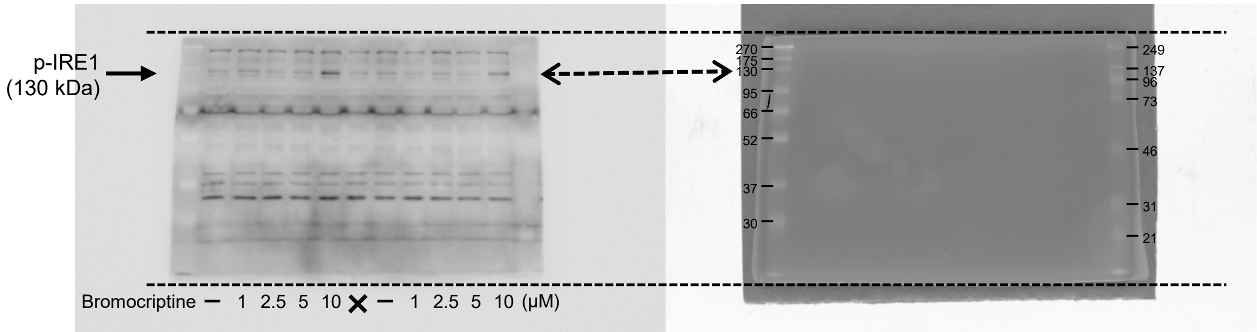

p-IRE1α Repeat-3

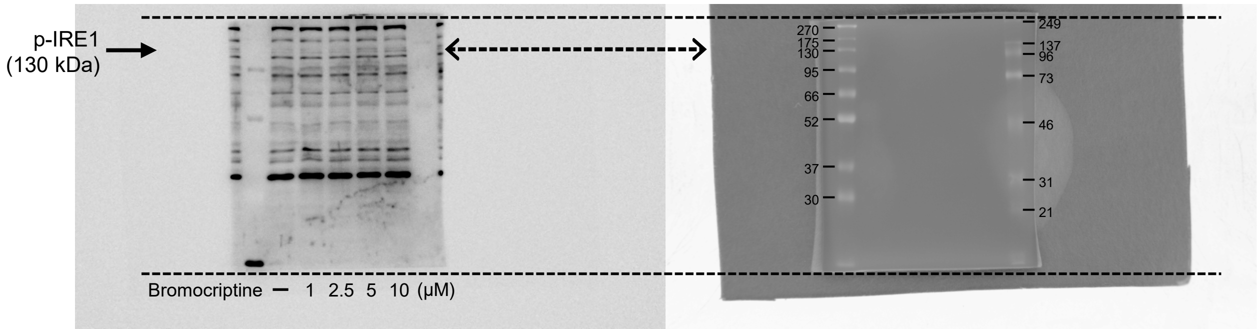

Capture method: ImageQuant LAS4000

Figure 7E: Western-blot raw data

Immunoblots

Molecular weight markers on the same gel

IRE1α Repeat-1

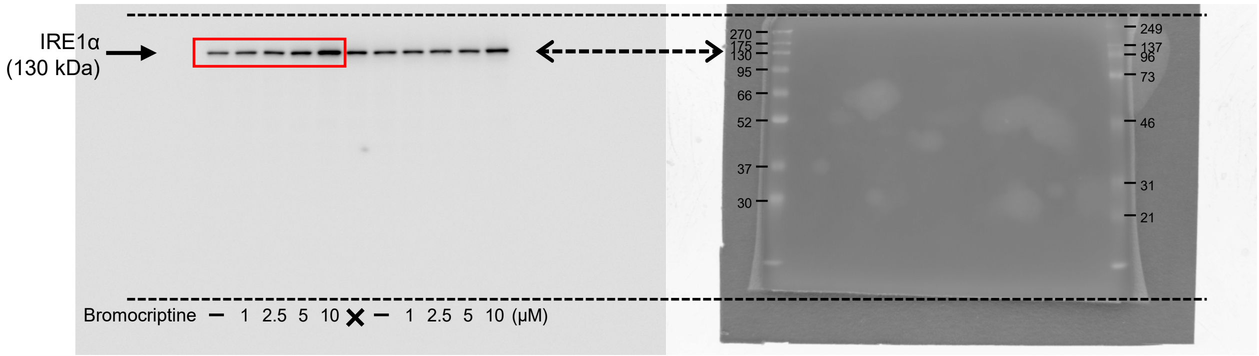

IRE1α Repeat-2

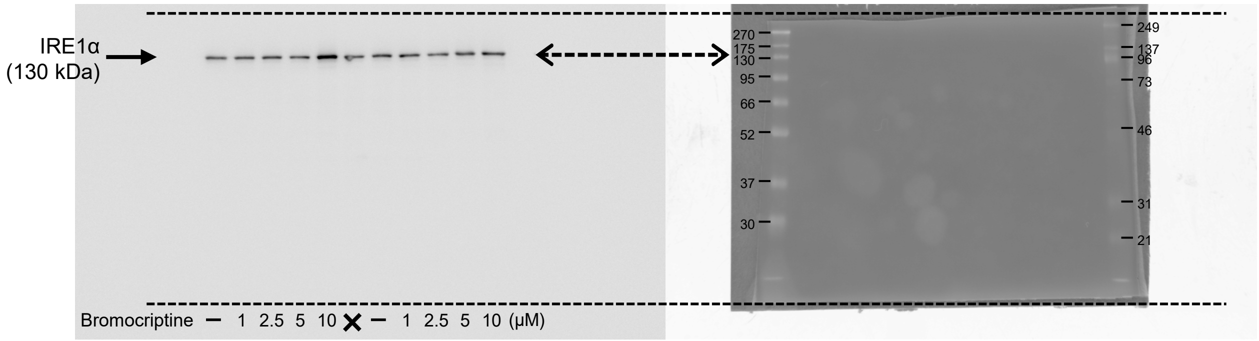

IRE1α Repeat-3

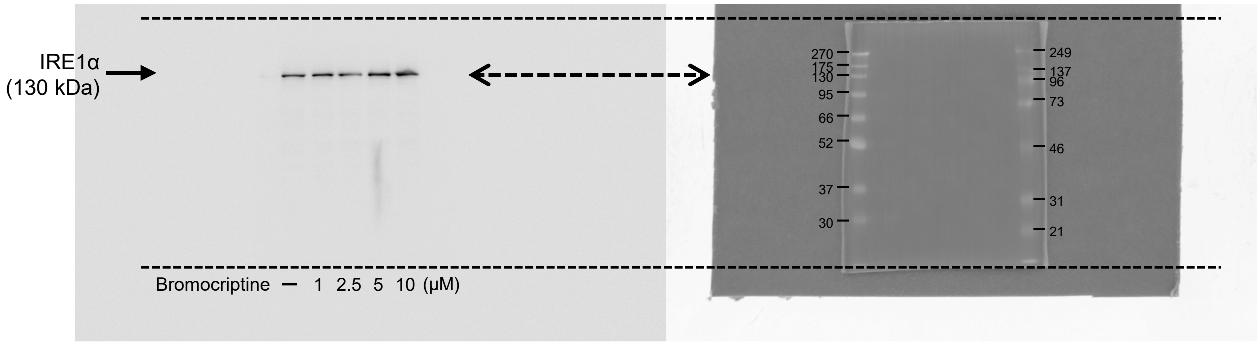

Capture method: ImageQuant LAS4000

Figure 7E: Western-blot raw data

Immunoblots | Molecular weight markers on the same gel

α-tubulin Repeat-1

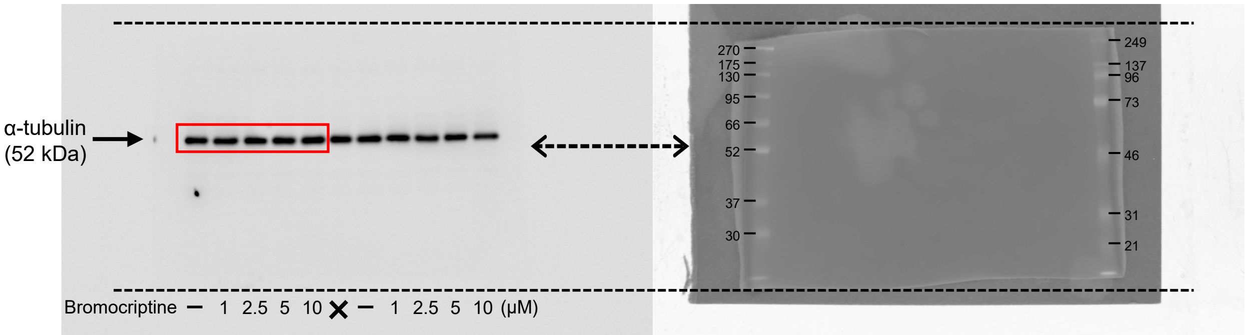

α-tubulin Repeat-2

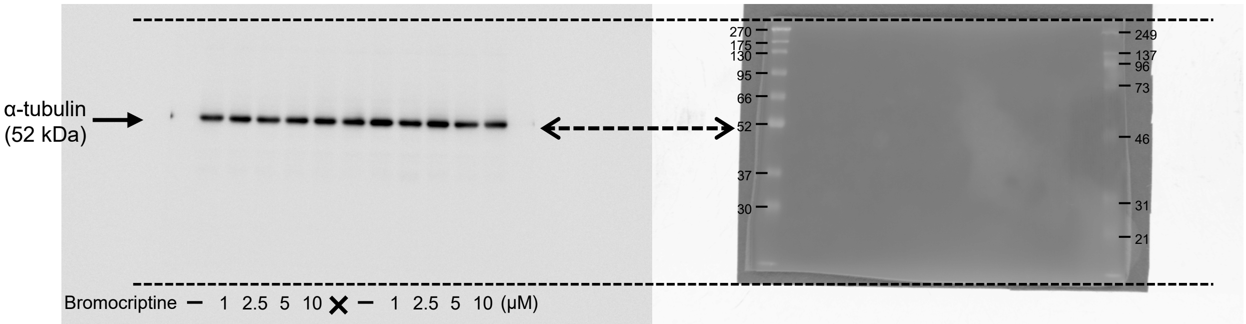

α-tubulin Repeat-3

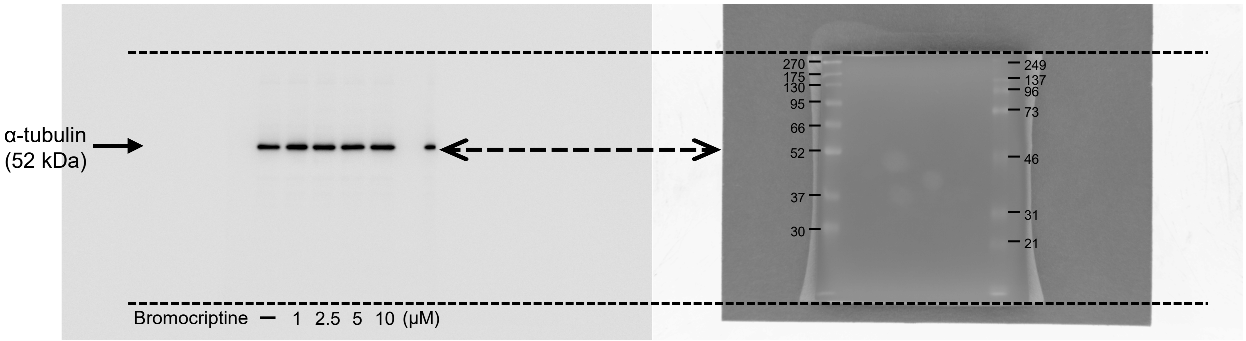

Capture method: ImageQuant LAS4000

Figure 7K: Western-blot raw data

Immunoblots | Molecular weight markers on the same gel

p-eIF2α Repeat-1

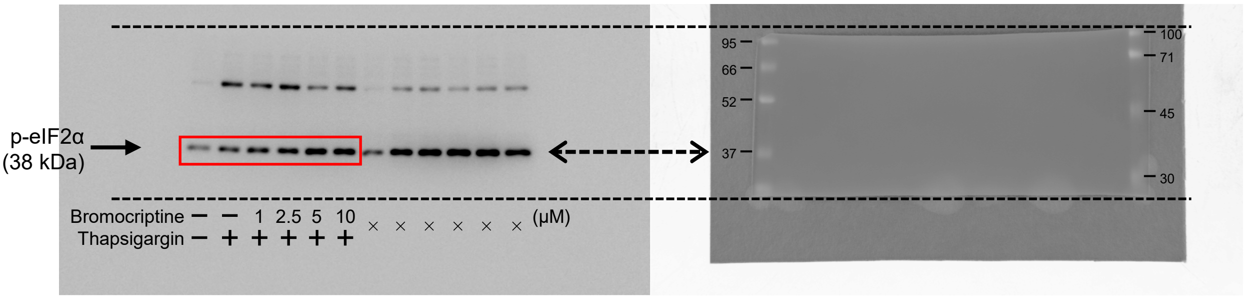

p-eIF2α Repeat-2

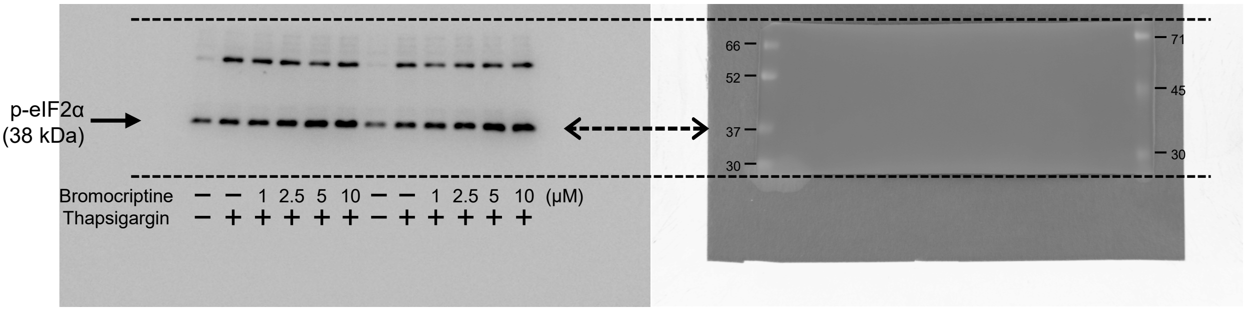

p-eIF2α Repeat-3

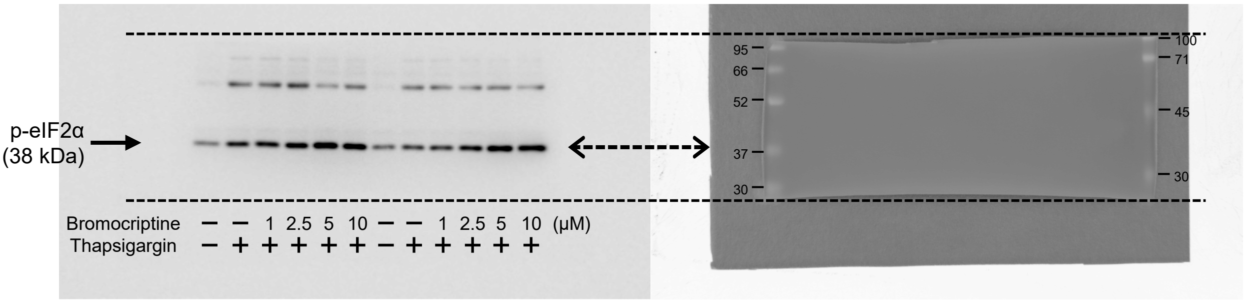

Capture method: ImageQuant LAS4000

Figure 7K: Western-blot raw data

Immunoblots

Molecular weight markers on the same gel

eIF2α Repeat-1

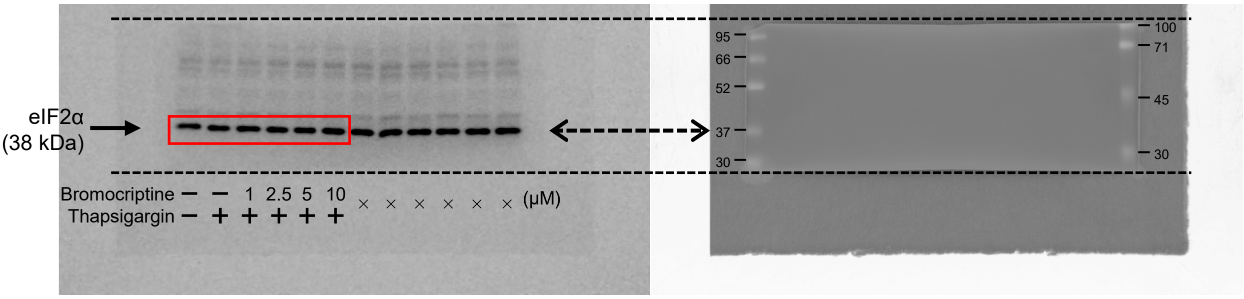

eIF2α Repeat-2

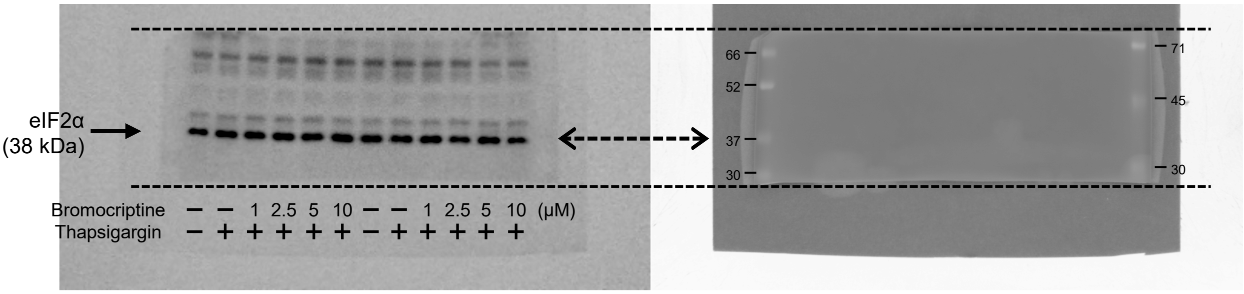

eIF2α Repeat-3

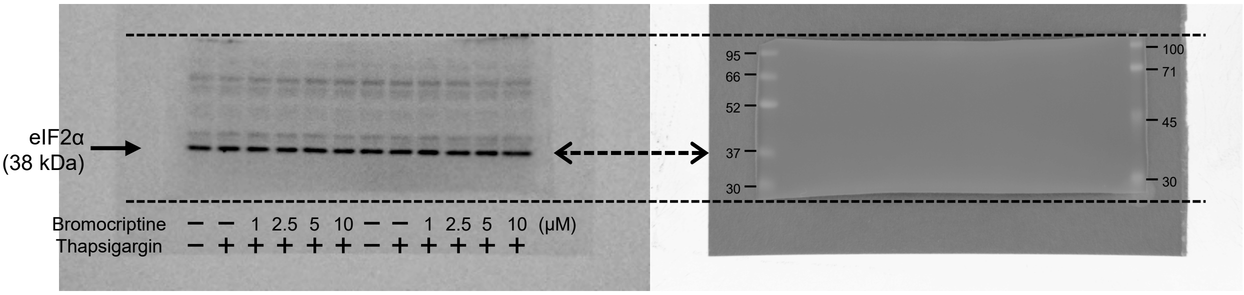

Capture method: ImageQuant LAS4000

Figure 7K: Western-blot raw data

Immunoblots

Molecular weight markers on the same gel

CHOP Repeat-1

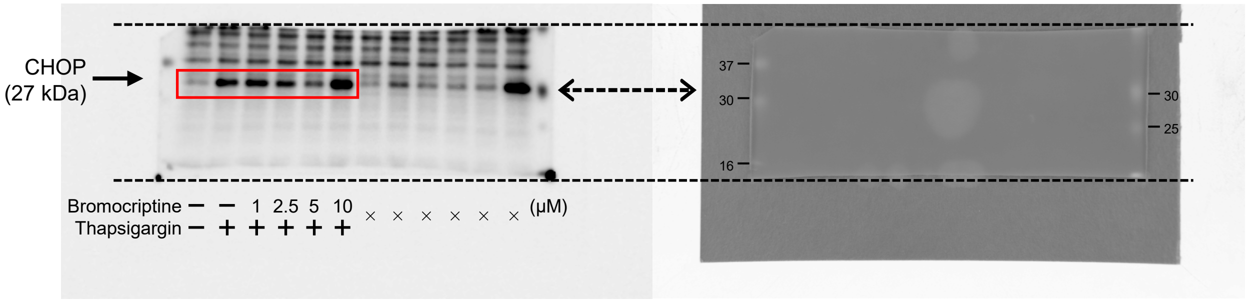

CHOP Repeat-2

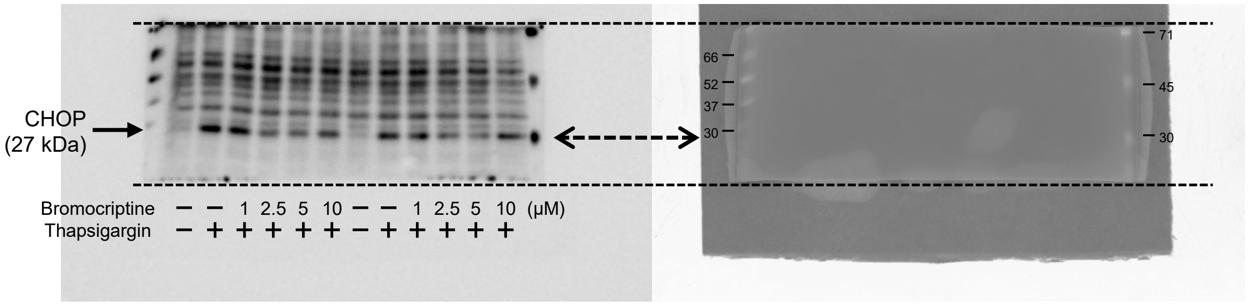

CHOP Repeat-3

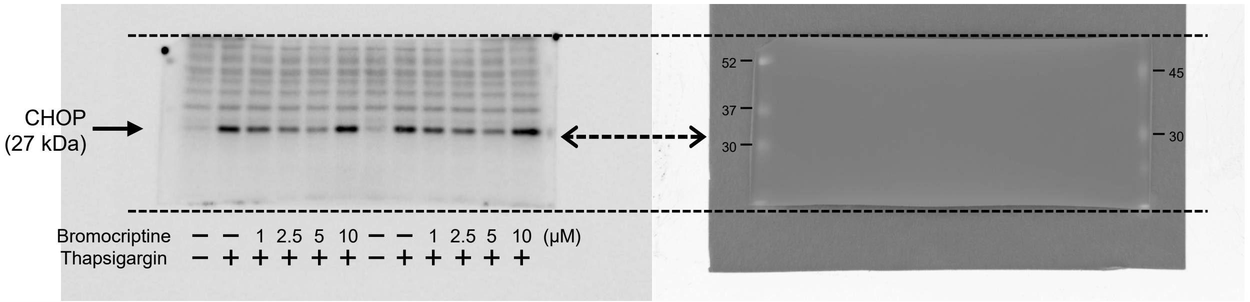

Capture method: ImageQuant LAS4000

Figure 7K: Western-blot raw data

Immunoblots | Molecular weight markers on the same gel

α-tubulin Repeat-1

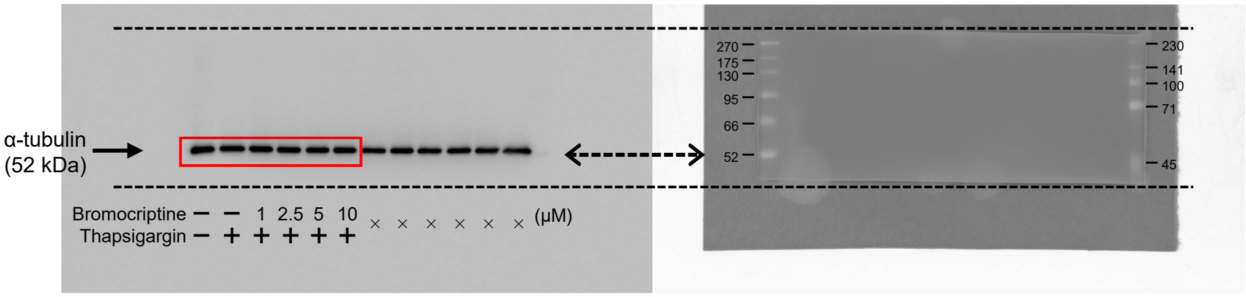

α-tubulin Repeat-2

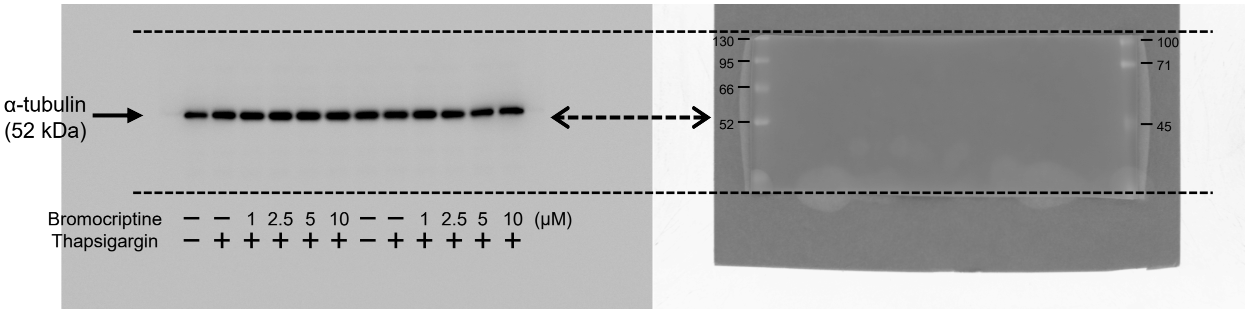

α-tubulin Repeat-3

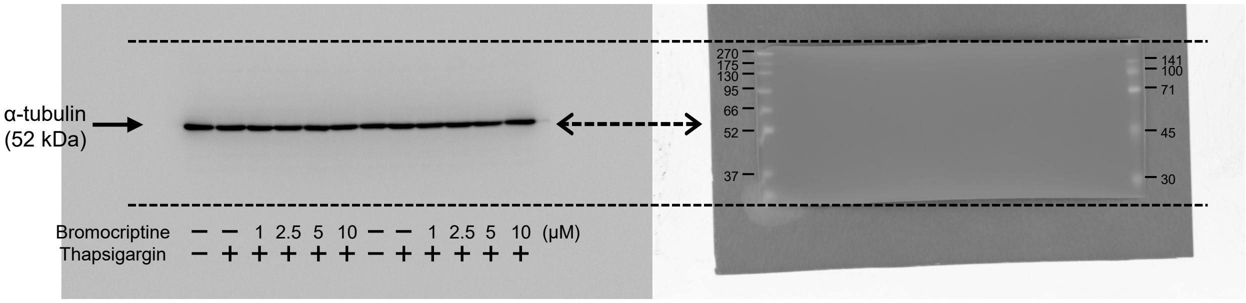

Capture method: ImageQuant LAS4000

S3 Fig: Western-blot raw data

Immunoblots

Molecular weight markers on the same gel

α-tubulin Repeat-1

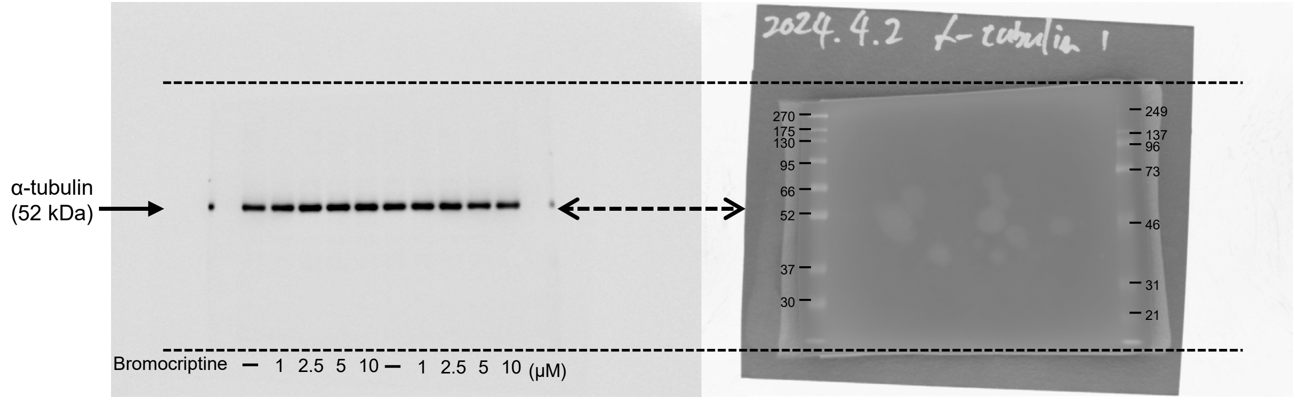

α-tubulin Repeat-2

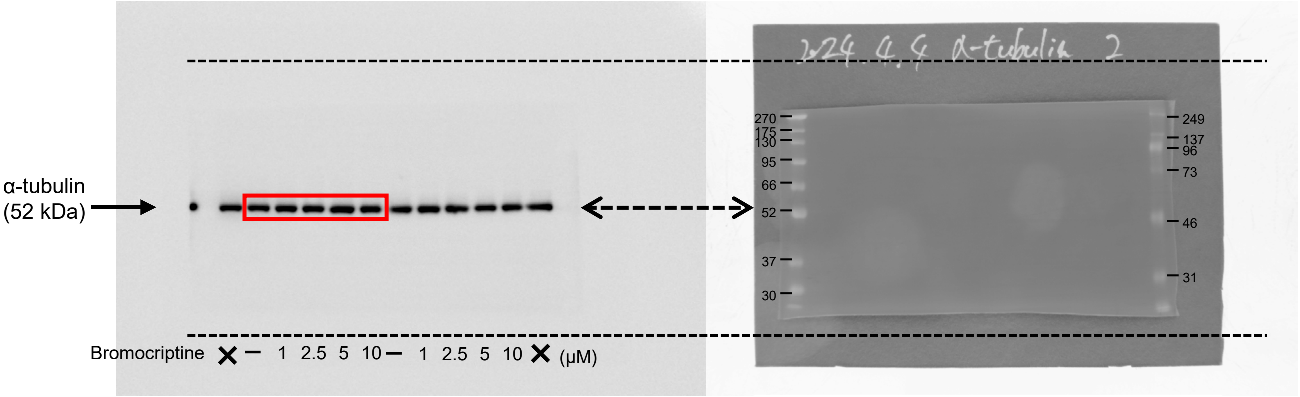

α-tubulin Repeat-3

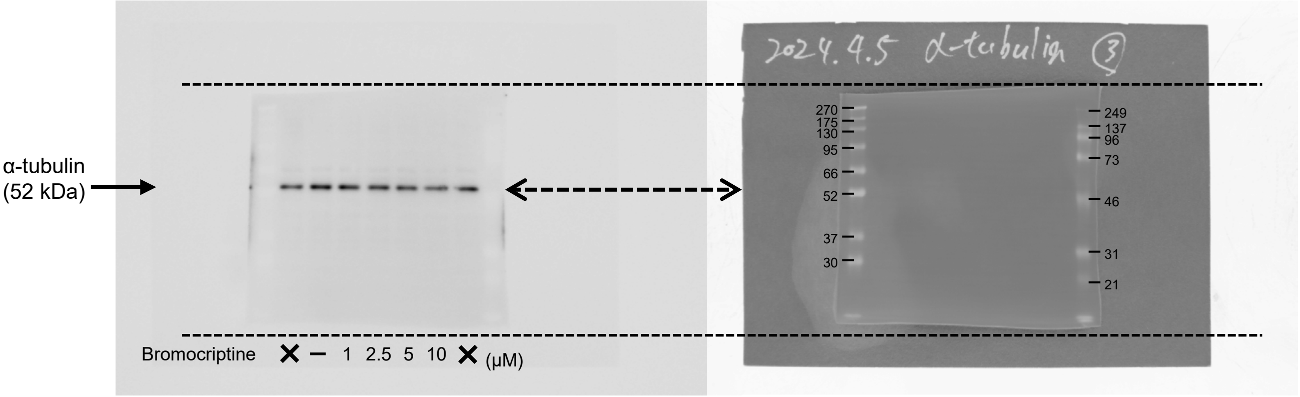

Capture method:
ImageQuant LAS4000

S3 Fig: Western-blot raw data

Immunoblots

Molecular weight markers on the same gel

PEPCK1 Repeat-1

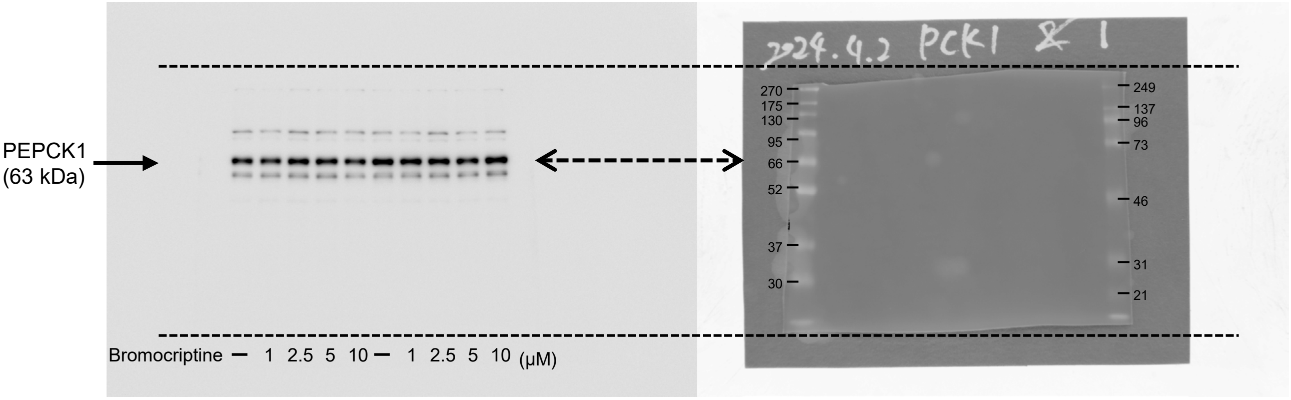

PEPCK1 Repeat-2

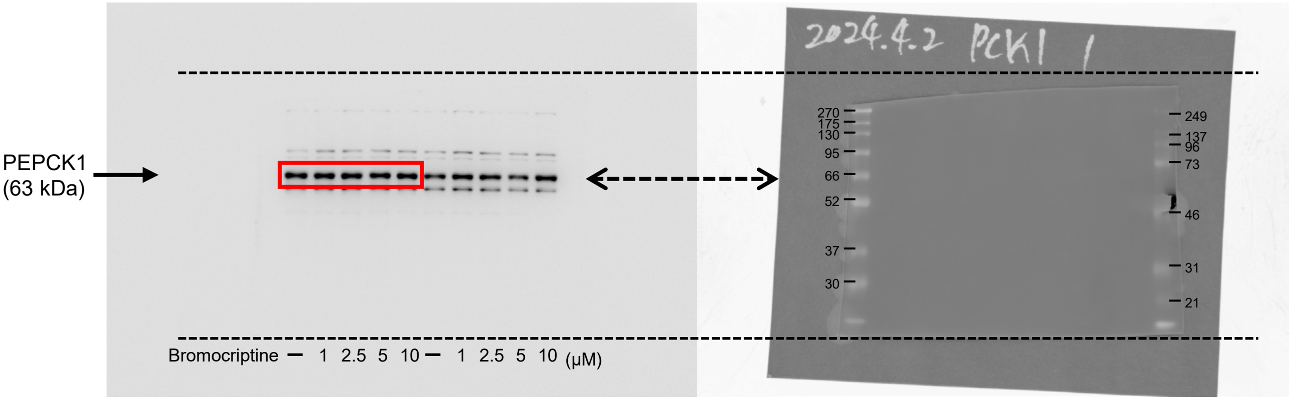

PEPCK1 Repeat-3

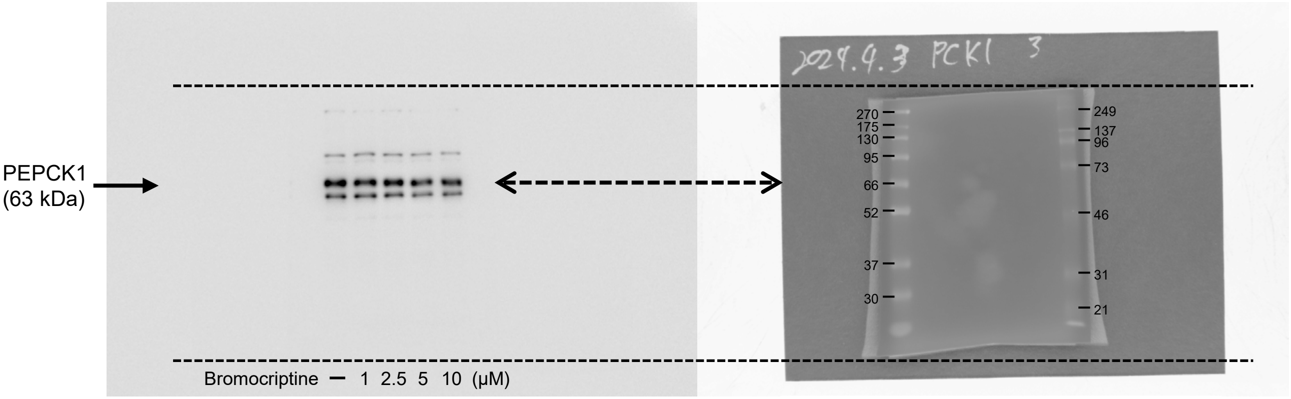

Capture method:
ImageQuant LAS4000

S4 Fig: Western-blot raw data

Immunoblots

Molecular weight markers on the same gel

β-Actin Repeat-1

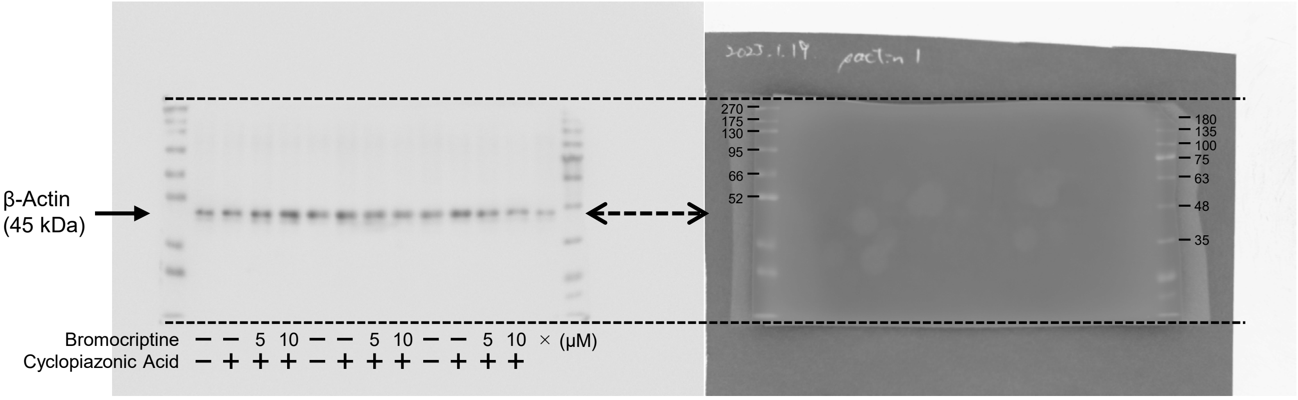

β-Actin Repeat-2

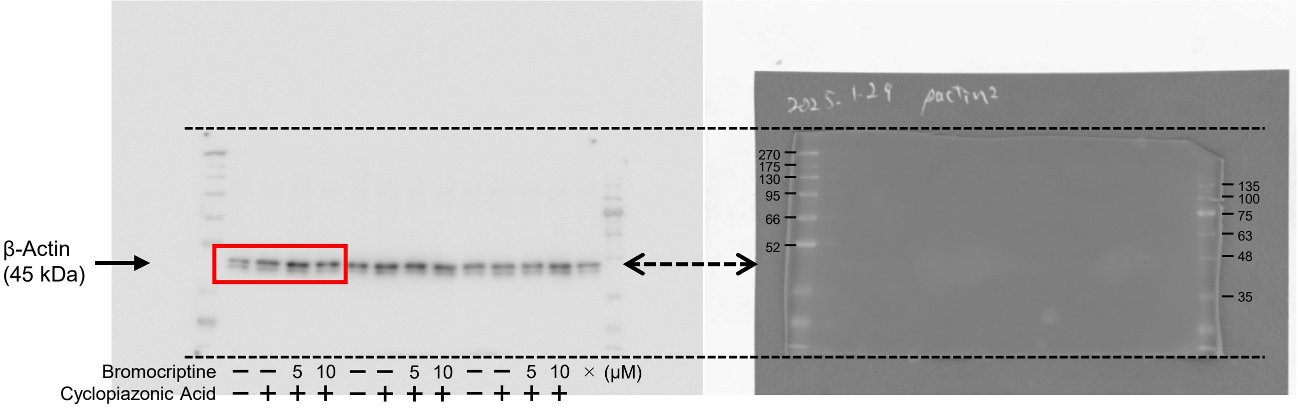

β-Actin Repeat-3

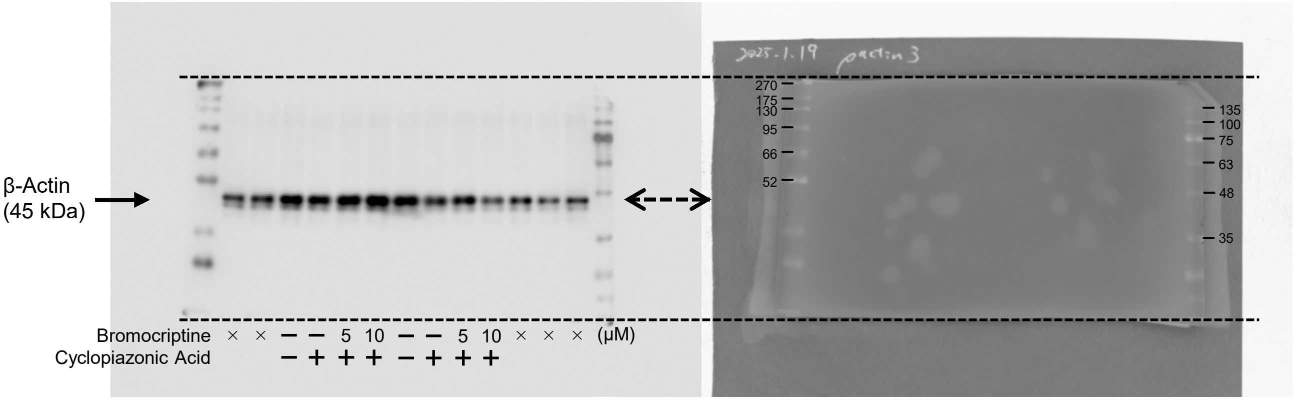

Capture method:
ImageQuant LAS4000

S4 Fig: Western-blot raw data

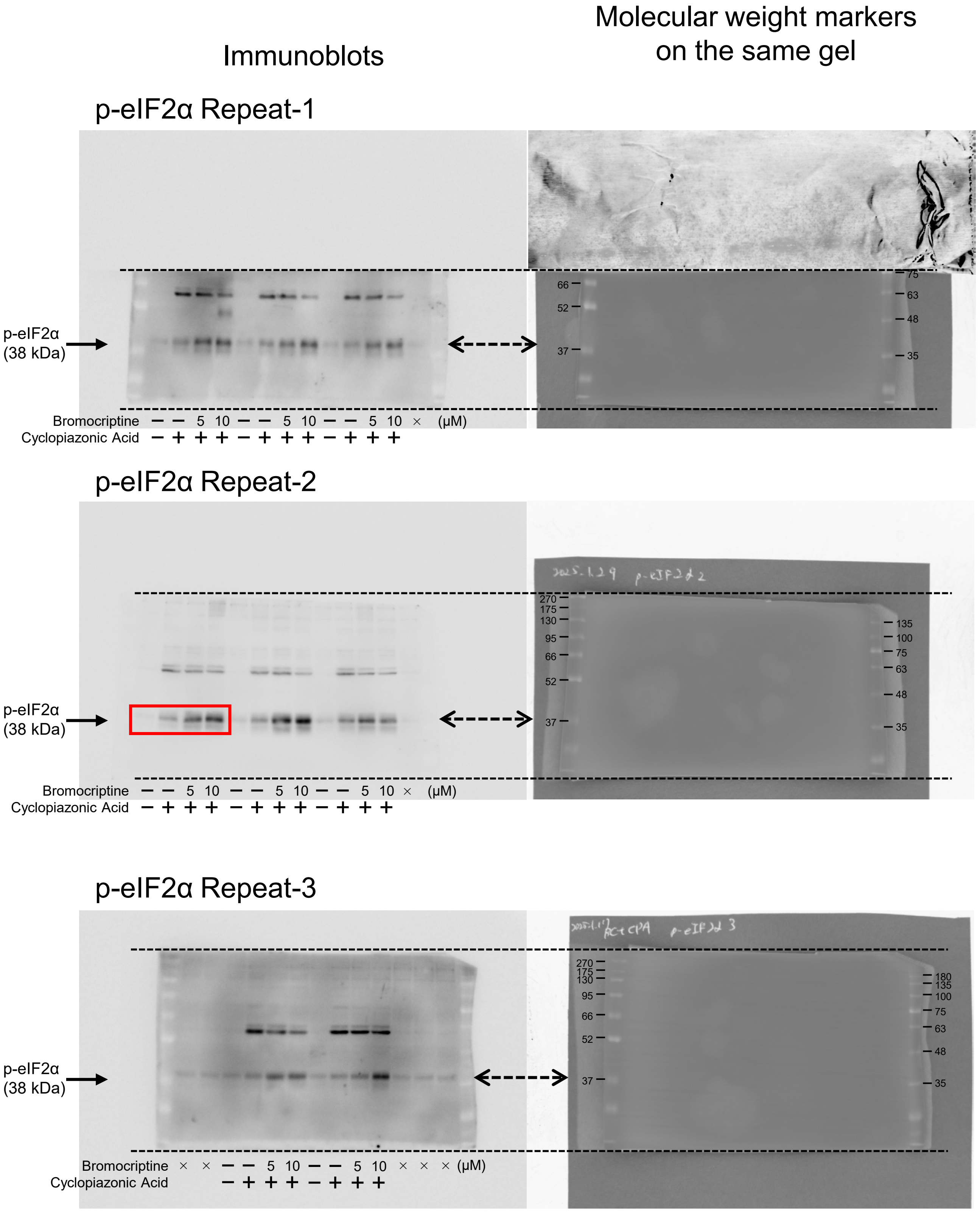

Capture method:
ImageQuant LAS4000

S4 Fig: Western-blot raw data

Immunoblots

Molecular weight markers on the same gel

eIF2α Repeat-1

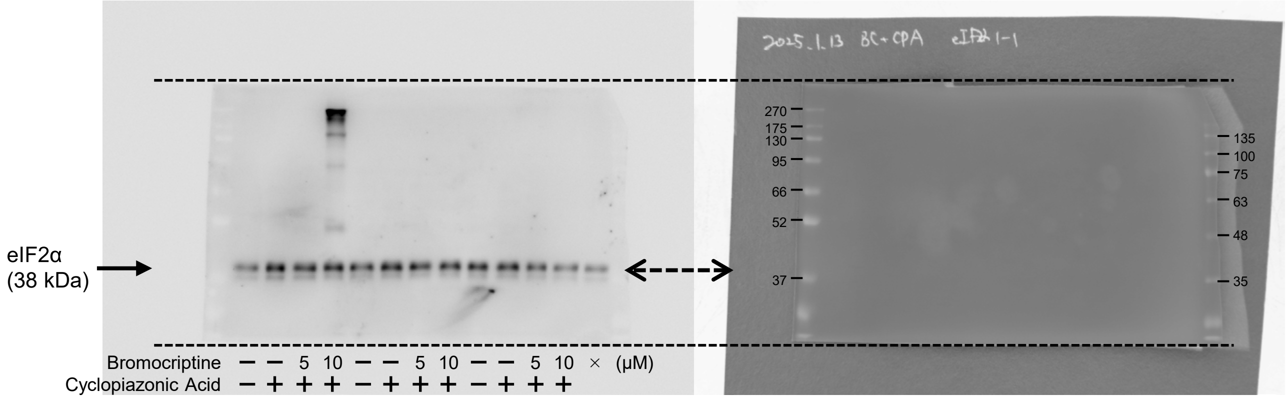

eIF2α Repeat-2

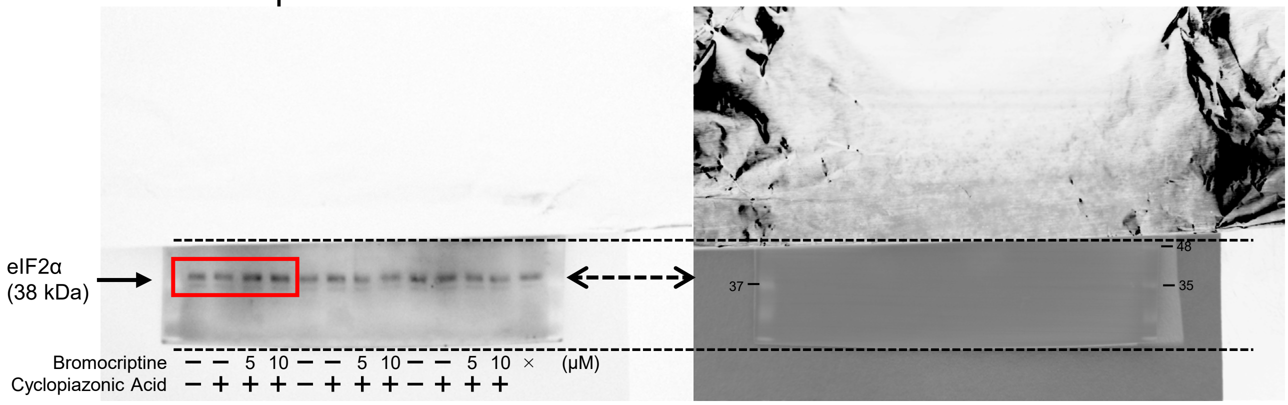

eIF2α Repeat-3

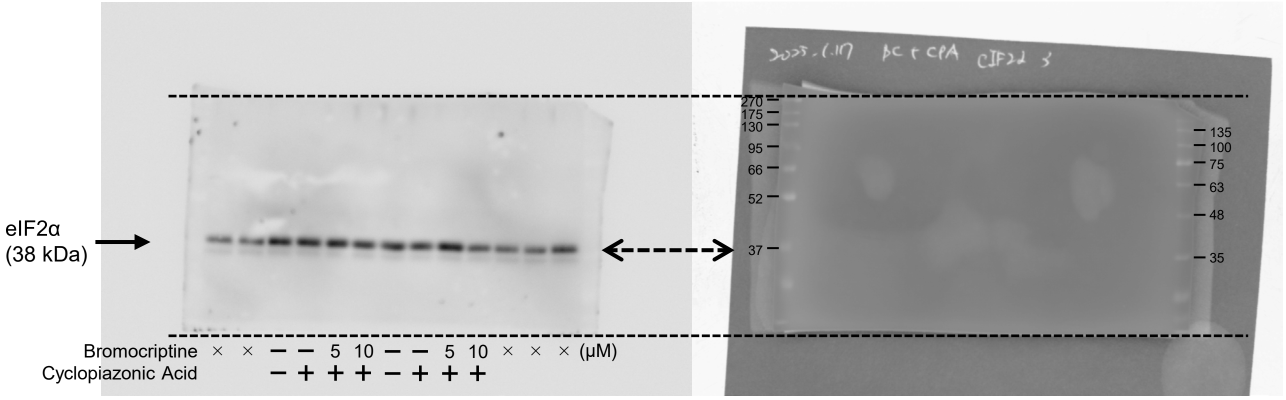

Capture method:
ImageQuant LAS4000

S4 Fig: Western-blot raw data

Immunoblots

Molecular weight markers on the same gel

CHOP Repeat-1

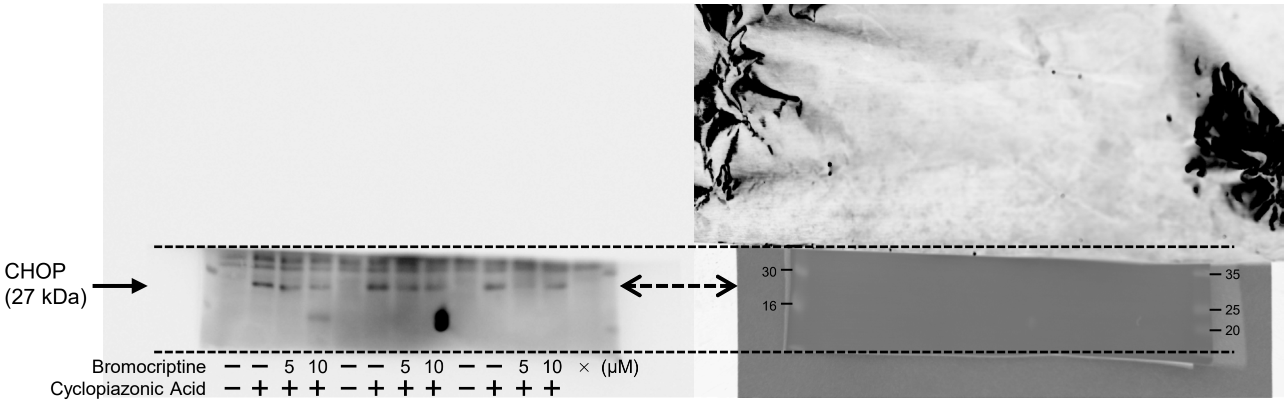

CHOP Repeat-2

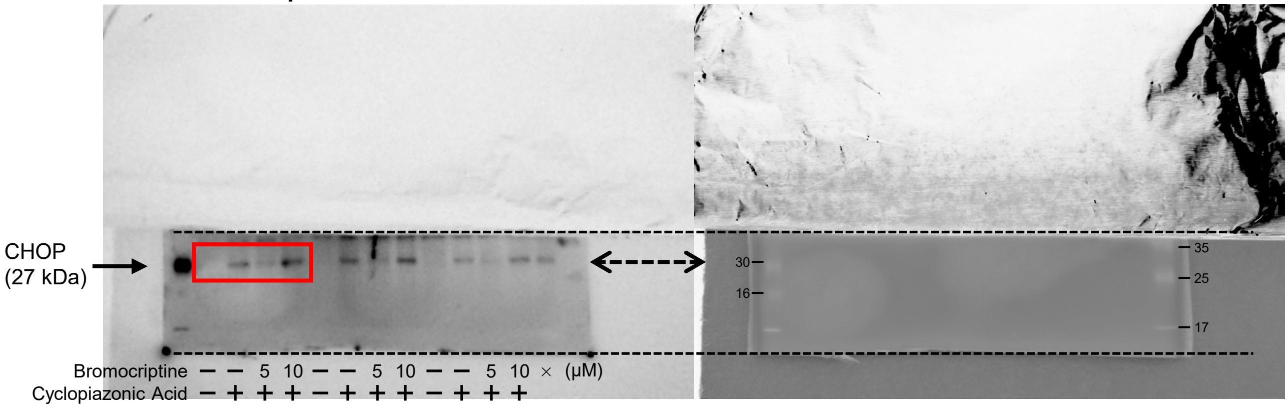

CHOP Repeat-3

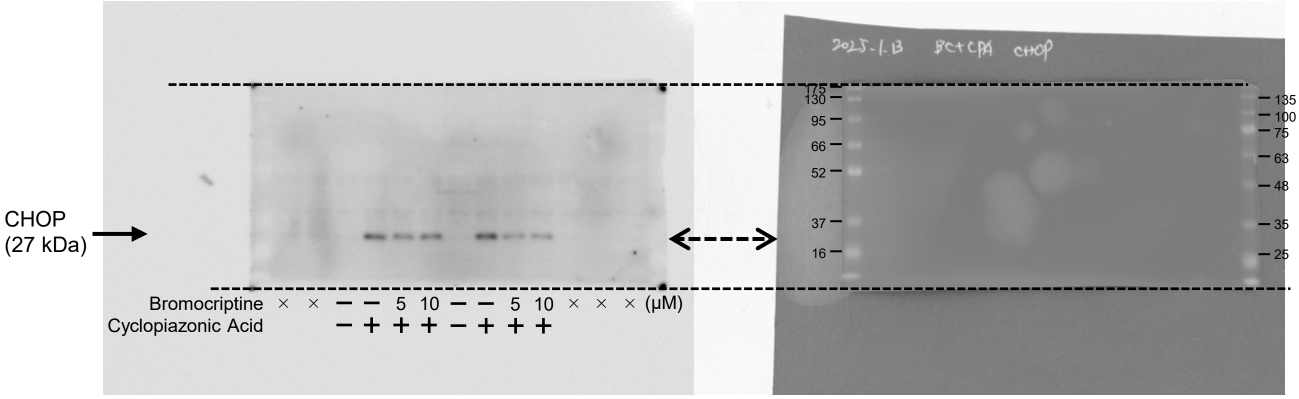

Capture method:
ImageQuant LAS4000
